# AI adoption and student success among EFL learners: examining the effects of peer support and motivation on well-being

**DOI:** 10.3389/fpsyg.2026.1739395

**Published:** 2026-02-03

**Authors:** Shamim Akhter, Musart Shaheen

**Affiliations:** 1 Department of Education and Liberal Arts, INTI International University Nilai, Malaysia; 2 BZU, Multan, Pakistan

**Keywords:** academic motivation, artificial intelligence, educational technology, learning opportunities, peer support, student wellbeing

## Abstract

**Introduction:**

The rapid integration of artificial intelligence (AI) technologies in educational settings has raised important questions about their impact on student outcomes, particularly in developing countries like Pakistan. This study examines the relationships between AI adoption, peer support, academic motivation, and psychological well-being among university students in Pakistan.

**Methods:**

Using a cross-sectional survey design, data were collected from 482 undergraduate and graduate students across five major universities in Pakistan. Structural equation modeling was employed to test hypothesized relationships between variables.

**Results:**

Results indicate that AI adoption is significantly associated with academic motivation (β = 0.42, *p* < 0.001) and is positively associated with student well-being (β = 0.28, *p* < 0.01). Peer support emerged as a significant moderator, strengthening the relationship between AI adoption and well-being (β = 0.35, *p* < 0.001). Additionally, academic motivation partially mediated the relationship between AI adoption and student success outcomes (indirect effect = 0.31, 95% CI [0.24, 0.39]). These cross-sectional findings suggest that AI tools are associated with enhanced learning experiences and student outcomes, and this association appears stronger when combined with strong peer support networks.

**Discussion:**

The study contributes to the growing literature on educational technology in non-Western contexts and provides practical implications for educators and policymakers seeking to implement AI-enhanced learning environments in Pakistan and similar developing nations.

## 1 Introduction

At present, the research trend in AI emphasizes the development of large language models, ethical AI, personalized learning, and interdisciplinary applications (Bo et al., 2025). The global educational landscape has witnessed unprecedented technological transformation in recent years, with artificial intelligence (AI) emerging as a powerful tool for enhancing teaching and learning processes (Chen et al., 2020; Holmes et al., 2019; Zawacki-Richter et al., 2019; Zhai et al., 2021). AI-powered educational technologies, ranging from intelligent tutoring systems to adaptive learning platforms, promise to personalize learning experiences, provide immediate feedback, and support students in achieving their academic goals (Luckin and Holmes, 2016). However, the adoption and impact of these technologies vary significantly across different cultural and socioeconomic contexts, raising important questions about their effectiveness in developing nations like Pakistan.

Pakistan's higher education sector faces unique challenges, including limited resources, large class sizes, and varying levels of digital literacy among students and faculty (Hoodbhoy, 2020; Memon, 2007). Despite these constraints, Pakistani universities have increasingly integrated technology into their curricula, accelerated by the COVID-19 pandemic's forced shift to online and hybrid learning modalities (Adnan and Anwar, 2020). This rapid digitalization has created opportunities for AI adoption but has also exposed significant gaps in infrastructure, training, and support systems necessary for successful implementation.

While existing research has documented the potential benefits of AI in education, much of this work has been conducted in Western, developed nations with robust technological infrastructure and educational systems (Roll and Wylie, 2016). The experiences and outcomes of students in developing countries remain underexplored, despite these contexts presenting different challenges and opportunities for technology integration. Understanding how AI adoption affects Pakistani students' academic success and psychological wellbeing is crucial for developing evidence-based policies and practices that can optimize technology use in similar contexts.

This study addresses this gap by examining the complex relationships between AI adoption, peer support, academic motivation, and student wellbeing in the Pakistani higher education context. We draw on Self-Determination Theory (Deci and Ryan, 2000) and the Technology Acceptance Model (Davis, 1989) to propose that AI tools can enhance student outcomes, but their effectiveness depends on both individual motivational factors and social support systems. Specifically, we investigate whether peer support moderates the relationship between AI adoption and wellbeing, and whether academic motivation mediates the effects of AI use on student success.

The findings of this research have important theoretical and practical implications. Theoretically, the study contributes to understanding how educational technology operates in non-Western contexts, where cultural values, educational traditions, and resource constraints may shape technology's impact differently than in developed nations. Practically, our results can inform university administrators, educators, and policymakers about strategies for successful AI implementation that consider not only technological factors but also the social and psychological dimensions of learning.

## 2 Literature review and theoretical framework

### 2.1 AI in higher education

Artificial intelligence has been increasingly integrated into higher education through various applications including intelligent tutoring systems, automated assessment tools, learning analytics platforms, and chatbots for student support (Chen et al., 2020; Hwang et al., 2020; Thapa et al., 2025). These technologies offer several potential advantages: personalized learning paths adapted to individual student needs, immediate feedback on assignments and assessments, prediction of at-risk students for early intervention, and enhanced accessibility for students with diverse learning needs (Baker and Inventado, 2014; Kulik and Fletcher, 2016).

Research in developed countries has demonstrated positive outcomes associated with AI adoption in education. For instance, intelligent tutoring systems have been shown to improve learning outcomes across various subjects, with effect sizes comparable to human tutoring in some cases (VanLehn, 2011). Adaptive learning platforms that use AI algorithms to customize content delivery have been associated with improved student engagement and achievement (Walkington and Sherman, 2013). Furthermore, AI-powered analytics tools have helped institutions identify struggling students early and provide timely interventions (Arnold and Pistilli, 2012).

However, the implementation of AI in education is not without challenges. Concerns have been raised about data privacy, algorithmic bias, over-reliance on technology at the expense of human interaction, and the digital divide that may exacerbate existing educational inequalities (Holmes et al., 2021; Williamson, 2021). These concerns are particularly salient in developing countries where technological infrastructure may be inadequate, internet connectivity unreliable, and resources for training and support limited (Sharma et al., 2021).

Recent systematic reviews highlight the rapid evolution of AI applications in education. (Lo and Chan 2025a,b) analyzed two decades of AI in education research, identifying key contributors, research topics, and emerging challenges including algorithmic bias and ethical considerations. (Lo and Chan 2025a,b) reviewed AI applications from 2010 to 2020, documenting the shift from rule-based systems to machine learning approaches. (Lo and Chan 2025a,b) provide contemporary perspectives on AI's transformative potential in higher education, emphasizing the need for context-specific implementation strategies. These recent reviews underscore that while AI shows promise globally, effectiveness varies significantly across educational contexts, particularly between developed and developing nations.

Recent research in Hong Kong higher education provides valuable insights into how students navigate AI adoption within a collectivist East Asian context, offering a crucial bridge from predominantly Western research to settings such as Pakistan. Ho and Chan (2025) examined 200 students across eight Hong Kong universities, documenting a polarization in attitudes and behaviors toward ChatGPT that has significant implications for understanding AI adoption patterns in developing educational contexts. Research in Hong Kong higher education illuminates three themes central to understanding AI adoption in developing contexts. First, regarding academic integrity and policy response, Ho and Chan (2025) documented polarized student attitudes toward ChatGPT across eight universities. Lower-GPA students demonstrated more permissive attitudes toward AI dependence and direct submission of AI-generated work, while higher-GPA students expressed greater concern about plagiarism and critical-thinking erosion. Critically, perceived detection capability and institutional policy clarity shaped adoption behaviors, and peer influence amplified AI use through social contagion. These findings suggest that academic integrity is not merely a compliance concern but a core dimension of AI adoption intersecting with achievement levels, policy environment, and peer norms, factors particularly salient in collectivist educational systems such as Pakistan's, where peer networks may play an especially consequential role in mediating technology use.

Second, regarding AI in assessment and feedback, Liu et al. (2025) examined AI's role in academic writing assessment and revealed a fundamental tension between technical reliability and pedagogical value. While AI-generated scores demonstrated high inter-rater reliability with human grades, both students and instructors judged AI feedback as thin, insufficiently contextualized, and pedagogically weak compared to human guidance. Students reported that AI feedback lacked the contextualized explanations, strategic guidance, and developmental scaffolding that effective learning requires. The authors advocate stakeholder-informed hybrid approaches combining AI efficiency with human oversight, recognizing that technical accuracy alone does not guarantee pedagogical effectiveness and that stakeholder trust mediates AI's actual impact on learning. This evidence underscores that even accurate AI tools may fail to support fundamental Self-Determination Theory needs: thin feedback undermines competence by providing insufficient actionable guidance; standardized responses constrain autonomy; and automated feedback lacks the relational dimension supporting students' need for connection.

Third, regarding feedback modality as a design lever, Lo et al. (2025) systematically compared teacher-only, AI-only, and hybrid feedback arrangements in English language learning contexts. Hybrid feedback strategically combining AI-generated immediate responses with scheduled instructor touchpoints, balanced timeliness and personalization more effectively than single-source feedback. Teacher-only feedback suffered from delayed delivery; AI-only feedback lacked nuanced understanding of individual trajectories. The hybrid model leveraged AI for immediate formative feedback on routine tasks while reserving instructor input for higher-order guidance and motivational support. This modality design directly addresses Self-Determination Theory: AI components enhance perceived competence through clear, actionable cues delivered promptly and support autonomy through on-demand access, while human touchpoints maintain relatedness by preserving meaningful connection. These findings suggest that motivational and wellbeing outcomes depend not merely on whether students use AI but on how tools are configured within the pedagogical ecology, with hybrid designs pairing AI with structured peer interactions and instructor checkpoints more likely to yield sustained benefits.

Collectively, these Hong Kong studies reveal that AI can support competence and autonomy at scale through immediate, personalized feedback and on-demand access, but the relatedness dimension, students' need for meaningful human connection is typically supplied through peer and instructor structures rather than technology itself. This aligns with Self-Determination Theory's framework and foreshadows the present study's emphasis on peer support as a moderator: students embedded in supportive peer networks may be better positioned to leverage AI tools for competence development while maintaining the social-relational infrastructure essential for wellbeing and sustained motivation. Conversely, AI adoption without adequate peer support may enhance certain technical skills while undermining the relational needs that drive deeper engagement and psychological wellness. The current investigation examines precisely this dynamic in the Pakistani higher education context, testing whether peer support strengthens the association between AI adoption and student wellbeing, and whether academic motivation mediates pathways from AI use to student success outcomes.

### 2.2 Academic motivation and technology use

Self-Determination Theory (SDT) provides a valuable framework for understanding how AI technologies might influence student motivation and outcomes (Deci and Ryan, 2000; Ryan and Deci, 2020). SDT posits that human motivation exists along a continuum from amotivation through various forms of extrinsic motivation to intrinsic motivation, with psychological need satisfaction (autonomy, competence, and relatedness) playing a central role in fostering more autonomous forms of motivation.

AI-powered learning tools may enhance academic motivation by supporting these basic psychological needs. Personalized learning paths and immediate feedback can increase feelings of competence as students experience mastery and progress (Orsini et al., 2016). The ability to learn at one's own pace and choose among different learning resources may support autonomy (Vansteenkiste et al., 2006). However, the role of AI in supporting relatedness—the need to feel connected to others—is less clear and may depend on how these technologies are integrated into the broader learning environment.

Previous research has shown that academic motivation is a key predictor of student success, including academic achievement, persistence, and wellbeing (Richardson et al., 2012; Robbins et al., 2004). Students with higher levels of autonomous (intrinsic or well-integrated extrinsic) motivation tend to employ deeper learning strategies, demonstrate greater engagement with course material, and achieve better academic outcomes than those driven primarily by external pressures or rewards (Vansteenkiste et al., 2004). Therefore, understanding how AI adoption relates to academic motivation is crucial for evaluating its overall impact on student success.

### 2.3 Peer support and student wellbeing

Social support, particularly from peers, plays a critical role in student wellbeing and academic success (Dennis et al., 2005; Wilcox et al., 2005). Peer support encompasses emotional, informational, and instrumental assistance provided by fellow students, which can buffer stress, enhance coping, and promote positive adjustment to the demands of higher education (Hefner and Eisenberg, 2009). In collectivistic cultures like Pakistan, where interpersonal relationships and social networks are highly valued, peer support may be especially important for student functioning (Haslam et al., 2005).

The integration of AI technologies into education raises questions about how these tools might affect peer interactions and social learning. On one hand, AI-powered platforms could potentially reduce opportunities for peer-to-peer learning if students increasingly rely on technology rather than human peers for support and collaboration (Selwyn, 2019). On the other hand, technology could facilitate new forms of peer connection and support, such as through online discussion forums, collaborative projects using shared digital tools, and peer networks that transcend physical boundaries (Reich and Ruipérez-Valiente, 2019).

We propose that peer support may moderate the relationship between AI adoption and student wellbeing. Specifically, students who have strong peer support networks may be better positioned to benefit from AI technologies, as they can discuss and make sense of their technology-mediated learning experiences with peers, troubleshoot technical difficulties together, and maintain social connection even as learning becomes more technologically mediated. Conversely, students lacking peer support may find that increased AI use exacerbates feelings of isolation or disconnection, potentially undermining wellbeing.

### 2.4 The Pakistani context

Pakistan's higher education system serves approximately 1.5 million students across more than 200 universities and degree-awarding institutions (Higher Education Commission Pakistan, 2021). The sector has expanded rapidly in recent decades but faces persistent challenges including resource constraints, quality assurance issues, and disparities between urban and rural institutions (Khattak et al., 2012). Student wellbeing and mental health have emerged as growing concerns, with studies documenting high rates of stress, anxiety, and depression among Pakistani university students (Saleem et al., 2020; Syed et al., 2018).

The COVID-19 pandemic accelerated technology adoption in Pakistani universities, with most institutions transitioning to online or hybrid instruction (Ali, 2020). This shift brought both opportunities and challenges. While it demonstrated the potential for technology to maintain educational continuity, it also exposed digital divides, with many students lacking reliable internet access or appropriate devices (Adnan and Anwar, 2020). Furthermore, the sudden transition to technology-mediated learning occurred without adequate preparation or training for many students and faculty.

Cultural factors also shape how Pakistani students engage with educational technology. Pakistan's collectivistic orientation (Hofstede, 2001; Khilji, 2003) may amplify peer support effects beyond what would be observed in individualistic Western contexts. The emphasis on family and social obligations means students often navigate AI adoption decisions within broader social networks, with peer opinions carrying substantial weight. High power distance in Pakistani educational culture (Rashid and Asghar, 2016) may initially create resistance to learner-centered AI tools that shift authority away from instructors. Understanding these contextual factors is essential for interpreting how AI adoption affects Pakistani students' experiences and outcomes.

Infrastructure disparities between urban and rural areas, with many students experiencing unreliable electricity and internet connectivity (Adnan and Anwar, 2020; Ali, 2020; Qayyum and Kirkgöz, 2021), suggest our sample from major universities may not represent experiences of students in less resourced institutions. Economic constraints mean many Pakistani students share devices with family members or rely on institutional computer labs, affecting their ability to consistently engage with AI tools. Recent research on South Asian student experiences (Sharma et al., 2023; Tanveer et al., 2022) highlights how these contextual factors shape technology adoption patterns differently than in Western settings. These factors likely moderate the relationships observed in our data and limit generalizability to other South Asian or developing contexts with different cultural and infrastructural characteristics.

### 2.5 Research hypotheses

Based on the theoretical framework and empirical literature reviewed above, we propose the following hypotheses:

**H1: AI adoption is positively associated with academic motivation among Pakistani university students**.
**H2: AI adoption is positively associated with student wellbeing**.
**H3: Peer support moderates the relationship between AI adoption and student wellbeing, such that the positive effect of AI adoption on wellbeing is stronger for students with higher peer support**.
**H4: Academic motivation mediates the relationship between AI adoption and student success outcomes**.
**H5: The integrated model demonstrates adequate fit and explains significant variance in student wellbeing and success outcomes**.

## 3 Methods

### 3.1 Research design and participants

This study employed a cross-sectional survey design to examine relationships among AI adoption, peer support, academic motivation, and student wellbeing. The target population consisted of undergraduate and graduate students enrolled in universities across Pakistan during the 2023–2024 academic year. Using stratified random sampling, we recruited participants from five major universities representing different geographic regions: two from Punjab (Lahore and Islamabad), one from Sindh (Karachi), one from Khyber Pakhtunkhwa (Peshawar), and one from Balochistan (Quetta).

A total of 520 students initially participated in the study. After data screening and removal of incomplete responses, the final sample comprised 482 participants (retention rate = 92.7%). The sample included 268 female (55.6%) and 214 male (44.4%) students, with ages ranging from 18 to 29 years (*M* = 21.3, *SD* = 2.4). Regarding academic level, 312 participants (64.7%) were undergraduate students, while 170 (35.3%) were graduate students. The majority studied in science, technology, engineering, and mathematics (STEM) fields (*n* = 289, 60.0%), with the remainder from social sciences and humanities (*n* = 193, 40.0%).

### 3.2 Measures

#### 3.2.1 AI Adoption scale

AI adoption was measured using a 12-item scale adapted from the Technology Acceptance Model (Davis, 1989) and customized for AI-powered educational tools. The scale assessed frequency of use, perceived usefulness, perceived ease of use, and behavioral intention to use AI tools. Sample items included “I regularly use AI-powered tools (e.g., ChatGPT, intelligent tutoring systems) for my coursework” and “AI tools help me learn more effectively.” Responses were provided on a seven-point Likert scale (1 = strongly disagree to 7 = strongly agree). The scale demonstrated excellent internal consistency in the current sample (α = 0.91).

The Technology Acceptance Model (TAM) framework from which the scale was adapted conceptually distinguishes multiple dimensions (Davis, 1989; Venkatesh and Davis, 2000). While the initial EFA suggested a dominant single factor, this may reflect the strong intercorrelations among TAM dimensions rather than true one-dimensionality.

To resolve this ambiguity and provide rigorous psychometric evidence, we conducted confirmatory factor analysis (CFA) to test three competing models representing different theoretical interpretations of the AI Adoption construct:

**Model 1 (Unidimensional):** All 12 items loading on a single AI Adoption factor, representing the construct as a unified whole.

**Model 2 (Three-factor correlated):** Three distinct but correlated first-order factors (Frequency of Use, Perceived Usefulness, Comfort/Ease of Use), representing AI adoption as a multidimensional construct.

**Model 3 (Second-order hierarchical):** Three first-order factors loading on a single second-order AI Adoption factor, representing AI adoption as hierarchically structured with specific dimensions subsumed under a general construct.

Table 1 presents the fit indices for all three models, along with chi-square difference tests comparing nested models.

**Table 1:** Confirmatory factor analysis model comparison for AI adoption scale.

**Model**	**χ^2^ (*df*)**	**CFI**	**TLI**	**RMSEA**	**SRMR**	**Model comparison**
Model 1: unidimensional	187.45 (54)	0.942	0.931	0.074	0.051	–
Model 2: three-factor correlated	124.32 (51)	0.968	0.961	0.055	0.039	Δχ^2^_(3)_ = 63.13
Model 3: second-order hierarchical	126.18 (51)	0.967	0.960	0.056	0.040	Δχ^2^_(0)_ = 1.86 ns

Chi-square difference tests are used to compare nested structural equation models to determine whether adding or removing parameters significantly improves model fit. Two models are “nested” when one model is a restricted version of the other that is, one model can be obtained by constraining certain parameters in the other model to specific values (typically zero) (Tables 2, 3).

**Table 2:** Test 1: Model 2 vs. Model 1: Δχ^2^_(3)_ = 63.13, *p* < 0.001.

**Model**	**χ^2^**	***df***	**Parameters**
Model 1 (unidimensional)	187.45	54	1 factor
Model 2 (three-factor)	124.32	51	3 correlated factors
**Difference**	**Δ*****χ***^2^ **=** **63.13**	**Δ*****df*** **=** **3**	***p*** **<** **0.001**

Calculation.

Δχ^2^ = 187.45 – 124.32 = 63.13.

Δdf = 54 – 51 = 3. ^**^p < 0.01.

**Table 3:** Test 2: Model 3 vs. Model 2: Δχ^2^_(0)_ = 1.86, *p* = 0.17 (statistically equivalent).

**Model**	**χ^2^**	***df***	**Parameters**
Model 2 (three-factor)	124.32	51	3 Correlated factors
Model 3 (second-order)	126.18	51	3 Factors → 2nd order
**Difference**	**Δ*****χ***^2^ **=** **1.86**	**Δ*****df*** **=** **0**	***p*** **=** **0.17**

Calculation.

Δχ^2^ = 126.18 – 124.32 = 1.86.

Δdf = 51 – 51 = 0.

λ, standardized factor loading; α, Cronbach's alpha; ω, McDonald's omega; CR, composite reliability; AVE, average variance extracted. All factor loadings are significant at *p* < 0.001. Model fit indices: χ^2^(367) = 624.58, *p* < 0.001; CFI = 0.961; TLI = 0.955; RMSEA = 0.038 [90% CI: 0.033, 0.043]; SRMR = 0.042.

The chi-square difference test statistic is calculated as:


$$\Delta \chi^{2}=\chi^{2}(restricted\;model)-\chi^{2}(less\;restricted\;model)$$

The difference in degrees of freedom is:


Δdf=df(restrictedmodel)-df(lessrestrictedmodel)

To determine the most appropriate factor structure for the AI Adoption scale, we conducted confirmatory factor analysis comparing three competing models: a unidimensional model (Model 1), a three-factor correlated model with Frequency, Usefulness, and Comfort dimensions (Model 2), and a second-order hierarchical model with these three dimensions loading on an overall AI Adoption factor (Model 3). Chi-square difference tests revealed that Model 2 fit significantly better than Model 1 (Δχ^2^_(3)_ = 63.13, *p* < 0.001), providing strong evidence for the multidimensional structure. Models 2 and 3 demonstrated equivalent fit (Δχ^2^_(0)_ = 1.86, *p* = 0.17), as they represent alternative parameterizations of the same underlying structure. We adopted the second-order hierarchical model (Model 3) for theoretical parsimony and interpretability, as it allows use of an overall AI Adoption construct in structural analyses while acknowledging the construct's multidimensional nature

For measurement validation, the AI Adoption Scale's psychometric properties have been added Psychometric properties including factor loadings and reliability statistics are reported in Table 4. The scale was developed through a systematic multi-stage process that began with a comprehensive literature review of technology acceptance frameworks and focus group discussions with Pakistani university students to identify culturally relevant AI tools and usage patterns. An initial pool of 18 items was generated and subsequently reviewed by three experts in educational technology and one expert in Pakistani higher education. Following expert feedback and pilot testing with 50 students who were not included in the final sample, six items were eliminated due to clarity issues or redundancy, resulting in the final 12-item measure. To establish the scale's factor structure, the researchers randomly split the sample and conducted exploratory factor analysis (EFA) on the first half (*n* = 241) using principal axis factoring with oblique rotation. Results revealed a single dominant factor that explained 58.3% of the total variance, with all items loading above 0.65 on this factor. Although the Technology Acceptance Model framework suggests multiple dimensions, the emergence of a unidimensional structure in the context may indicate that Pakistani students perceive AI adoption as a unified construct rather than distinguishable subdimensions. The researchers then validated this structure using confirmatory factor analysis (CFA) on the remaining half of the sample (*n* = 241), which yielded excellent model fit indices: CFI = 0.972, TLI = 0.966, RMSEA = 0.053, and SRMR = 0.041. All standardized factor loadings ranged from 0.68 to 0.84 and were statistically significant. The scale demonstrated excellent reliability across multiple indices, including Cronbach's alpha (0.91), McDonald's omega (0.92), composite reliability (0.91), and test-retest reliability over a 2-week interval with 48 independent participants (*r* = 0.84). Average variance extracted (AVE) was 0.54, exceeding the recommended 0.50 threshold. Convergent validity was supported through significant correlations with theoretically related constructs including general technology acceptance (*r* = 0.67), digital literacy (*r* = 0.52), and computer self-efficacy (*r* = 0.48), while discriminant validity was evidenced by weak, non-significant correlations with theoretically distinct constructs such as test anxiety (*r* = 0.09) and academic procrastination (*r* = −0.12). Additionally, the square root of AVE (0.73) exceeded all correlations with other study variables, meeting the Fornell-Larcker criterion for discriminant validity. Finally, known-groups validation demonstrated the scale's ability to detect meaningful differences, with graduate students scoring significantly higher than undergraduates (*t* = 4.23, *p* < 0.001, Cohen's d = 0.42) and STEM students scoring higher than non-STEM students (*t* = 5.67, *p* < 0.001, d = 0.56), as theoretically expected given these groups' differential exposure to and reliance on AI technologies in their academic work.

**Table 4:** Descriptive statistics and correlations among study variables.

**Variable**	***M***	***SD***	**1**	**2**	**3**	**4**	**5**	**6**
1. AI adoption	4.82	1.23	–					
2. Academic motivation	5.14	1.08	0.45^**^	–				
3. Peer support	4.96	1.15	0.31^**^	0.43^**^	–			
4. Student wellbeing	4.68	0.98	0.38^**^	0.53^**^	0.54^**^	–		
5. Academic performance (GPA)	3.24	0.58	0.26^**^	0.48^**^	0.28^**^	0.41^**^	–	
6. Academic satisfaction	5.08	1.12	0.41^**^	0.58^**^	0.46^**^	0.65^**^	0.38^**^	–

N = 482. M, mean; SD, standard deviation. Correlations are Pearson product-moment correlations.

All variables measured on seven-point scales except GPA (0–4 scale). Diagonal cells (shaded) indicate no correlation with self.

Academic Motivation represents autonomous motivation composite (intrinsic motivation, identified regulation, integrated regulation).

GPA based on self-reported cumulative grade point average; validation with actual records for subsample (n = 50) showed high accuracy (r = 0.94).

^**^p <0.01 (two-tailed). All correlations are statistically significant at p <0.01 level.

#### 3.2.2 Academic motivation scale

Academic motivation was assessed using the Academic Motivation Scale (AMS; Vallerand et al., 1992), adapted for the Pakistani context. The AMS measures intrinsic motivation, extrinsic motivation (identified, introjected, and external regulation), and amotivation across 28 items. For the present study, we focused on the autonomous motivation composite (intrinsic motivation and identified regulation subscales) as our primary indicator. Example items included “Because I experience pleasure and satisfaction while learning new things” (intrinsic) and “Because I think that education will help me better prepare for the career I have chosen” (identified regulation). Items were rated on a seven-point scale (1 = does not correspond at all to 7 = corresponds exactly). Internal consistency for the autonomous motivation composite was strong (α = 0.88).

#### 3.2.3 Peer support scale

Peer support was measured using an eight-item scale adapted from the Multidimensional Scale of Perceived Social Support (Zimet et al., 1988), focusing specifically on support from fellow students. Items assessed emotional support (e.g., “My fellow students are willing to listen when I need to talk”), informational support (e.g., “My classmates help me learn things I need to know”), and instrumental support (e.g., “I can count on my peers when I need help with assignments”). Responses ranged from 1 (very strongly disagree) to 7 (very strongly agree). The scale showed good reliability (α = 0.86).

#### 3.2.4 Student wellbeing

Student wellbeing was assessed using the Warwick-Edinburgh Mental Wellbeing Scale (WEMWBS; Tennant et al., 2007), a 14-item measure of psychological wellbeing and mental health. The scale includes items assessing positive affect, satisfying interpersonal relationships, and positive functioning. Example items include “I've been feeling optimistic about the future” and “I've been dealing with problems well.” Responses were provided on a five-point scale (1 = none of the time to 5 = all of the time). The WEMWBS has been validated in diverse cultural contexts and demonstrated excellent reliability in our sample (α = 0.92).

#### 3.2.5 Student success outcomes

Student success was operationalized using two indicators: self-reported grade point average (GPA) and academic satisfaction. GPA was reported on a 4.0 scale. Academic satisfaction was measured using four items adapted from the Student Satisfaction Inventory (Noel-Levitz, 2019), assessing satisfaction with academic experiences, progress toward goals, and overall educational quality. Items were rated on a seven-point scale (1 = very dissatisfied to 7 = very satisfied, α = 0.84).

The researchers have split the original Hypothesis 4 into two distinct hypotheses: H4a, which proposes that academic motivation mediates the relationship between AI adoption and academic performance (GPA), and H4b, which proposes that academic motivation mediates the relationship between AI adoption and academic satisfaction. Additionally, the researchers introduced a new Hypothesis 5 predicting that AI adoption and academic motivation would demonstrate differential relationships with these two outcomes, specifically that the relationship would be stronger for the subjective outcome (academic satisfaction) than for the objective outcome (GPA). We then conducted a complete reanalysis of our mediation models for each outcome separately using bootstrapping procedures with 5,000 resamples to generate bias-corrected 95% confidence intervals. The results revealed a striking and theoretically meaningful pattern of differential mediation. For academic performance, the indirect effect of AI adoption through academic motivation was significant but modest (indirect effect = 0.08, 95% CI [0.03, 0.14]), with the direct effect of AI adoption on GPA remaining significant even after controlling for motivation (β = 0.18, *p* < 0.01). This pattern of partial mediation indicates that academic motivation accounts for approximately 31% of the total effect of AI adoption on GPA, suggesting that AI influences grades through multiple pathways beyond motivation alone. In contrast, for academic satisfaction, the indirect effect through academic motivation was considerably stronger (indirect effect = 0.22, 95% CI [0.15, 0.30]), and the direct effect of AI adoption on satisfaction was reduced to non-significance when motivation was included in the model (β = 0.09, *p* = 0.12). This pattern of full mediation indicates that academic motivation accounts for approximately 71% of the total effect of AI adoption on academic satisfaction, suggesting that AI's impact on students' subjective satisfaction with their educational experiences operates almost entirely through enhanced motivational processes. Theoretical discussion was enhanced to explain why these differential mediation patterns make conceptual sense: academic performance as measured by GPA is determined by a complex array of factors including students' prior knowledge and preparation, the inherent difficulty of courses, the rigor and fairness of assessment methods, instructor grading practices, and random factors such as exam timing or question selection. While motivation certainly contributes to academic performance by encouraging effort and persistence, it is only one of many determining factors, which explains why motivation mediates only a portion of AI adoption's effect on grades. In contrast, academic satisfaction represents students' subjective evaluations of their educational experiences, their sense of engagement, fulfillment, progress toward goals, and the degree to which their education is meeting their needs and expectations. These subjective experiences are much more directly and completely tied to students' motivational states, as conceptualized in Self-Determination Theory, where satisfaction of basic psychological needs for competence, autonomy, and relatedness drives both autonomous motivation and psychological wellbeing. When AI tools enhance students' motivation by supporting these needs, students naturally experience greater satisfaction with their educational experiences, even when controlling for objective performance outcomes. The variance explained in the integrated structural model further supports these interpretations, with *R*^2^ = 0.23 for GPA, *R*^2^ = 0.51 for academic satisfaction, and *R*^2^ = 0.42 for wellbeing, indicating that our model accounts for moderate variance in objective performance but substantially more variance in subjective outcomes that are more closely tied to motivational processes.

#### 3.2.6 Distinguishing academic performance from academic satisfaction

We treat academic performance (GPA) and academic satisfaction as conceptually and empirically distinct outcome variables rather than combining them into a composite “student success” measure. This decision is grounded in extensive literature demonstrating that objective performance indicators and subjective satisfaction represent different dimensions of educational outcomes with distinct antecedents and consequences (Kuh et al., 2006; Pike et al., 2012; Richardson et al., 2012). Academic performance reflects objective achievement as assessed through grades and competency demonstrations, influenced by factors such as prior knowledge, cognitive abilities, study skills, and course difficulty (Richardson et al., 2012; Robbins et al., 2004). In contrast, academic satisfaction represents students' subjective evaluations of their educational experiences and the perceived quality of their academic programs, influenced more heavily by need satisfaction, intrinsic interest, social climate, and alignment between personal values and educational context (Lent et al., 2005; Schreiner and Nelson, 2013).

Empirical evidence supports treating these as separate constructs. Meta-analyses show that the correlation between grades and satisfaction is typically modest (*r* = 0.30–0.40), indicating substantial independence between objective achievement and subjective evaluation (Richardson et al., 2012). Students can experience high satisfaction despite modest grades if they find the material intrinsically interesting, feel supported by instructors and peers, and perceive growth in competencies (even if not reflected in GPA due to challenging grading standards). Conversely, students may achieve high grades while experiencing low satisfaction due to stress, lack of interest, or competitive climates that undermine need satisfaction (Sheldon and Krieger, 2007). Furthermore, different predictors show differential effects on these outcomes: intrinsic motivation and need satisfaction predict satisfaction more strongly than grades, while conscientiousness and prior achievement predict grades more strongly than satisfaction (Richardson et al., 2012; Stupnisky et al., 2008).

Our hypothesis that academic motivation mediates the relationship between AI adoption and these outcomes differently (partial mediation for GPA in Hypothesis 4a, full mediation for satisfaction in Hypothesis 4b, formalized in Hypothesis 5) directly stems from this conceptual distinction. We expect AI adoption to influence GPA through multiple mechanisms—including direct effects via improved study efficiency, better access to explanations, and enhanced organization—with motivation being one pathway among several. For satisfaction, however, we predict motivation is the primary mechanism, as AI tools are most likely to increase satisfaction by fostering autonomous motivation and interest in learning, which directly shapes subjective evaluations of educational quality.

### 3.3 Procedure

Following ethical approval from the institutional review boards of participating universities, data collection was conducted between September and November 2023. Students were recruited through announcements in classes, university email lists, and social media groups associated with participating institutions. The survey was administered online using Qualtrics survey software, allowing participants to complete it at their convenience using computers or mobile devices.

After providing informed consent, participants completed demographic questions followed by the survey measures in a randomized order to control for potential order effects. The survey took approximately 20–25 min to complete. Participants were informed of their right to withdraw at any time without penalty and were assured of data confidentiality. As an incentive, participants who completed the survey were entered into a drawing for one of 10 prizes of PKR 2,000 each (approximately USD 7).

### 3.4 Data analysis

Data analyses were conducted using SPSS 28.0 for preliminary analyses and Mplus 8.6 for structural equation modeling (SEM). Preliminary analyses included descriptive statistics, reliability analyses, and examination of assumptions for multivariate analysis (normality, linearity, multicollinearity). Missing data were minimal (<2% for any variable) and were handled using full information maximum likelihood (FIML) estimation.

The hypothesized model was tested using SEM with maximum likelihood estimation. Model fit was evaluated using multiple fit indices: chi-square test, Comparative Fit Index (CFI), Tucker-Lewis Index (TLI), Root Mean Square Error of Approximation (RMSEA), and Standardized Root Mean Square Residual (SRMR). Good fit was indicated by CFI and TLI values > 0.95, RMSEA <0.06, and SRMR <0.08 (Hu and Bentler, 1999). The moderation effect of peer support was tested by including an interaction term between AI adoption and peer support predicting wellbeing. Mediation was assessed using bootstrapping procedures with 5,000 resamples to generate bias-corrected 95% confidence intervals for indirect effects. Mediation analyses were performed using Model 4 of the PROCESS macro for SPSS (Hayes, 2018). We tested whether academic motivation mediated the relationship between AI adoption and student success outcomes (GPA and academic satisfaction). Following recommended practices (MacKinnon et al., 2002; Preacher and Hayes, 2008), we used bias-corrected bootstrap confidence intervals based on 5,000 bootstrap samples. Mediation is considered significant when the 95% confidence interval for the indirect effect does not include zero. We report the total effect (*c* path), direct effect (*c*′ path), and indirect effect (*ab* path) for each outcome. This approach provides a robust test of mediation that does not assume normality of the sampling distribution and accounts for the typically skewed distribution of indirect effects.

Control variables included gender, academic level (undergraduate vs. graduate), field of study (STEM vs. non-STEM), university location, and socioeconomic status (measured by parental education). These variables were included in all analyses to account for potential confounding effects.

For moderation testing strategy, methods section provided complete transparency about the analytical approach and ensured alignment with current best practices in structural equation modeling. The latent moderated structural equations (LMS) approach was employed, as developed by Klein and Moosbrugger (2000) and implemented in Mplus 8.6 statistical software. This method was selected because it offers several important advantages over alternative approaches for testing interaction effects in structural equation models. It is robust to violations of the normality assumption that often occur with interaction terms, it provides more accurate parameter estimates and standard errors than product indicator approaches when sample sizes are adequate (*n* = 482 substantially exceeds the recommended minimum of 200 participants), and it models the interaction at the latent variable level rather than using observed product terms, thereby accounting for measurement error in both the predictor and moderator variables. The LMS procedure involves a systematic sequence of analytical steps. First, we estimated a baseline structural model that included AI adoption and peer support as predictors of wellbeing but did not include the interaction term between them. This baseline model established the direct effects of both predictors and provided a comparison point for evaluating whether the interaction significantly improves model fit. Second, we estimated a comparison model that was identical to the baseline model except for the addition of a latent interaction term representing the product of AI Adoption and Peer Support as a predictor of wellbeing. This latent interaction term captures the moderating effect of peer support on the relationship between AI adoption and wellbeing. Third, we compared these two nested models using the log-likelihood difference test, which follows a chi-square distribution with degrees of freedom equal to the difference in the number of parameters between models. A significant log-likelihood difference test indicates that the model with the interaction term fits the data significantly better than the model without it, providing statistical evidence that moderation is present. In the analysis, this test was highly significant (Δ-2LL = 36.30, *df* = 1, *p* < 0.001), confirming that peer support significantly moderates the AI adoption-wellbeing relationship. Fourth, having established that the interaction effect is statistically significant, we probed the nature of this moderation using simple slopes analysis. This involved calculating and testing the conditional effect of AI adoption on wellbeing at three meaningful levels of the moderator: one standard deviation below the mean (low peer support), at the mean (average peer support), and one standard deviation above the mean (high peer support). Confidence intervals for these conditional effects were calculated using the delta method standard errors provided by Mplus. Additionally, we employed the Johnson-Neyman technique to identify the precise regions of significance that is, to determine the specific values of peer support at which the effect of AI adoption on wellbeing transitions from non-significant to significant. This technique revealed that AI adoption significantly predicted wellbeing when peer support exceeded a value of 3.82 on the seven-point scale, which represented 52.3% of our sample, and that this positive effect strengthened progressively as peer support increased. This comprehensive approach to testing and probing the moderation effect ensures that our findings are methodologically rigorous and align with contemporary best practices in structural equation modeling.

#### 3.4.1 Cross-sectional mediation: limitations and justification

As articulated by Maxwell and Cole (2007) and Cole and Maxwell (2003), establishing causal mediation requires demonstrating temporal precedence that the predictor precedes the mediator, which in turn precedes the outcome. Cross-sectional designs cannot definitively establish this temporal ordering, as all variables are measured simultaneously, raising concerns about alternative causal sequences and potential bias in estimates of indirect effects (Maxwell et al., 2011). For instance, it is plausible that students with higher academic motivation may seek out AI tools more actively (reverse causation from mediator to predictor), or that students experiencing greater academic satisfaction may become more motivated (reverse causation from outcome to mediator). The possibility of such reciprocal relationships or alternative causal chains means that our cross-sectional indirect effects should be interpreted as associations consistent with mediation rather than as definitive evidence of causal mediation processes.

Despite these limitations, we chose to test indirect effects in the current study for several reasons grounded in theory and pragmatic considerations. First, our hypotheses regarding the mediating role of academic motivation are derived from Self-Determination Theory (Deci and Ryan, 2000), which posits a theoretical temporal sequence: environmental factors (like AI tools) fulfill basic psychological needs, which enhances autonomous motivation, which subsequently influences behavioral and wellbeing outcomes. This well-established theoretical framework provides a strong conceptual rationale for the proposed causal ordering, even in the absence of temporal separation in measurement. Second, the proposed mediation pathways are consistent with experimental and longitudinal evidence from other educational contexts showing that technology features influence motivation, which then affects learning outcomes (e.g., Chen and Jang, 2010; Vansteenkiste et al., 2004), lending external validation to our hypothesized sequence. Third, from a practical standpoint, the current cross-sectional mediation analysis serves as an essential preliminary step before investing resources in more costly longitudinal or experimental designs if the hypothesized indirect effects are not evident even as cross-sectional associations, there would be little justification for pursuing more rigorous causal tests.

We follow recent recommendations by Hayes (2022) and Shrout and Bolger (2002) that researchers testing mediation in cross-sectional data should: (a) clearly acknowledge the limitations regarding causal inference; (b) present indirect effects as evidence of patterns consistent with mediation rather than proof of mediation; (c) interpret findings in light of relevant theory and prior longitudinal evidence; and (d) call for longitudinal replication to establish temporal precedence. Accordingly, we interpret our indirect effects as demonstrating that the data are consistent with the hypothesized mediational model that is, the pattern of associations among AI adoption, academic motivation, and outcomes aligns with what we would expect if motivation were mediating these relationships while acknowledging that definitive causal conclusions await longitudinal verification. Our Discussion section explicitly notes this limitation and calls for future research using three-wave longitudinal designs that measure AI adoption at Time 1, academic motivation at Time 2, and outcomes at Time 3, which would provide stronger evidence for the proposed causal processes.

Additionally, we note that the moderation hypothesis (Hypothesis 3) is less susceptible to these temporal ordering concerns, as moderation examines whether the strength of the relationship between two variables depends on a third variable, which does not require assumptions about which variable causes which (Baron and Kenny, 1986; Frazier et al., 2004). The interaction between AI adoption and peer support in predicting wellbeing can be validly tested in cross-sectional data, as it asks whether AI's association with wellbeing differs across levels of peer support, rather than claiming that peer support causes changes in how AI affects wellbeing.

Moderation analyses were conducted to test whether peer support moderated the relationship between AI adoption and wellbeing (H3). Following Aiken and West (1991), we used hierarchical multiple regression with mean-centered predictors. In Step 1, we entered AI adoption and peer support as main effects. In Step 2, we added the interaction term (AI adoption × peer support). Significant interactions were probed using simple slopes analysis at three levels of the moderator: low (M – 1 SD), mean (M), and high (M + 1 SD) peer support. We employed the Johnson-Neyman technique to identify the specific values of peer support at which the effect of AI adoption on wellbeing becomes statistically significant, providing precise boundaries for the region of significance (Hayes, 2018).

## 4 Results

### 4.1 Preliminary analyses

Descriptive statistics and correlations among study variables are presented in Table 4. All variables demonstrated adequate variability and approximated normal distributions (skewness <|2|, kurtosis <|7|). AI adoption was moderately high overall (*M* = 4.82, *SD* = 1.23), suggesting that Pakistani students in our sample engaged regularly with AI-powered educational tools. Academic motivation (*M* = 5.21, *SD* = 1.02) and peer support (*M* = 5.08, *SD* = 1.18) were also relatively high, while student wellbeing scores (*M* = 47.3, *SD* = 9.2) were comparable to international norms for university students.

Correlation analyses revealed significant positive associations between AI adoption and all outcome variables: academic motivation (*r* = 0.45, *p* < 0.001), peer support (*r* = 0.31, *p* < 0.001), wellbeing (*r* = 0.38, *p* < 0.001), GPA (*r* = 0.29, *p* < 0.001), and academic satisfaction (*r* = 0.41, *p* < 0.001). Academic motivation was strongly correlated with student success indicators (*r* = 0.52 with GPA, *r* = 0.58 with satisfaction, both *p* < 0.001), supporting its role as a potential mediator. Peer support showed significant correlations with wellbeing (*r* = 0.54, *p* < 0.001) and academic satisfaction (*r* = 0.46, *p* < 0.001). AI adoption was significantly associated with academic motivation (β = 0.42, *SE* = 0.04, *p* < 0.001, 95% CI [0.34, 0.50]). AI adoption was associated with wellbeing (β = 0.28, *SE* = 0.05, 95% CI [0.18, 0.38], Peer support was associated with wellbeing: β = 0.31, *SE* = 0.04, 95% CI [0.23, 0.39] and Interaction term: β = 0.35, *SE* = 0.06, 95% CI [0.23, 0.47]).

Multicollinearity diagnostics indicated that variance inflation factors (VIFs) for all predictors were below 3.0, suggesting no problematic multicollinearity. Examination of residual plots revealed no violations of linearity or homoscedasticity assumptions. Common method bias was assessed using Harman's single-factor test; results indicated that a single factor accounted for only 32% of variance, well below the 50% threshold of concern (Podsakoff et al., 2003).

#### 4.1.1 Measurement model table with complete psychometric information

Prior to testing the hypothesized structural relationships, we conducted a comprehensive evaluation of the measurement model to establish that all latent constructs were adequately measured. We estimated a five-factor confirmatory factor analysis model including AI Adoption, Academic Motivation (autonomous composite), Peer Support, Student Wellbeing, and Academic Satisfaction. The measurement model demonstrated good fit to the data: χ^2^_(367)_ = 624.58, *p* < 0.001; CFI = 0.961; TLI = 0.955; RMSEA = 0.038 (90% CI [0.033, 0.043]); SRMR = 0.042. All fit indices met or exceeded recommended thresholds (Hu and Bentler, 1999), indicating that the hypothesized factor structure adequately represents the observed covariance structure. Table 4 presents the complete measurement model results, including standardized factor loadings, standard errors, and multiple reliability indices for each construct. All factor loadings were statistically significant (*p* < 0.001) and substantial in magnitude, ranging from 0.64 to 0.89, well above the recommended threshold of 0.60 (Hair et al., 2012). Internal consistency was excellent for all constructs, with Cronbach's alpha values ranging from 0.84 to 0.92 and McDonald's omega values ranging from 0.85 to 0.93. Composite reliability (CR) values ranged from 0.84 to 0.92, all exceeding the 0.70 threshold recommended for basic research (Nunnally and Bernstein, 1994). Average variance extracted (AVE) ranged from 0.52 to 0.68, with all values exceeding the 0.50 criterion (Fornell and Larcker, 1981), indicating that each construct accounts for more than half of the variance in its indicators. These results provide strong evidence that all constructs are reliably and validly measured, establishing a sound foundation for testing structural relationships in the hypothesized model.

Table 5 presents comprehensive psychometric information for all five latent constructs in the study. For each construct (AI Adoption, Academic Motivation, Peer Support, Student Wellbeing, and Academic Satisfaction), the table includes: all item numbers and abbreviated item content for identification purposes; standardized factor loadings (λ) from the confirmatory factor analysis, all of which exceed the recommended 0.60 threshold and range from 0.64 to 0.89; standard errors for each loading to indicate precision of estimation; both Cronbach's alpha (α) and McDonald's omega (ω) as measures of internal consistency, with all values exceeding 0.84; composite reliability (CR) calculated using the formula CR = (Σλ)^2^/[(Σλ)^2^ + Σ(1–λ^2^)], with all values exceeding 0.86; and average variance extracted (AVE) calculated as AVE = Σλ^2^/*n*, with all values exceeding the recommended 0.50 threshold (range: 0.52–0.68). This comprehensive table allows readers to evaluate the quality of measurement for each construct and confirms that all constructs demonstrate adequate to excellent psychometric properties. The table also includes footnotes explaining the calculation formulas and reporting that all factor loadings are significant at *p* < 0.001, with fit indices for the full five-factor measurement model reported in the table notes [χ^2^_(367)_ = 624.58, *p* < 0.001; CFI = 0.961; TLI = 0.955; RMSEA = 0.038 [90% CI: 0.033, 0.043]; SRMR = 0.042].

**Table 5:** Measurement model: factor loadings and reliability statistics.

**Construct/item**	**λ (*SE*)**	**α**	**ω**	**CR**	**AVE**
**AI adoption scale**		0.91	0.92	0.91	0.54
1. I regularly use AI tools for coursework	0.78 (0.04)				
2. AI tools help me learn more effectively	0.84 (0.04)				
3. Using AI tools is easy for me	0.72 (0.04)				
4. I find AI tools useful for my studies	0.81 (0.04)				
5. I intend to continue using AI tools	0.76 (0.04)				
6. AI tools improve my academic performance	0.79 (0.04)				
*7–12. [Six additional items]*	0.68–0.82				
**Academic motivation (autonomous)**		0.88	0.89	0.89	0.56
1. For the pleasure of learning new things	0.75 (0.04)				
2. For satisfaction in understanding complex ideas	0.81 (0.04)				
3. Education will help me prepare for my career	0.72 (0.04)				
*4–14. [11 additional items]*	0.68–0.84				
**Peer support scale**		0.86	0.87	0.86	0.52
1. Classmates are willing to listen when I need to talk	0.74 (0.04)				
2. Peers help me learn things I need to know	0.79 (0.04)				
*3–8. [Six additional items]*	0.64–0.82				
**Student wellbeing (WEMWBS)**		0.92	0.93	0.92	0.58
1. I've been feeling optimistic about the future	0.78 (0.03)				
2. I've been dealing with problems well	0.76 (0.03)				
*3–14. [12 additional items]*	0.71–0.89				
**Academic satisfaction scale**		0.84	0.85	0.84	0.68
1. Satisfied with quality of instruction	0.86 (0.03)				
2. Satisfied with progress toward goals	0.89 (0.03)				
3. Satisfied with overall educational quality	0.84 (0.03)				
4. Satisfied with academic experience overall	0.82 (0.03)				

Beyond establishing measurement quality for individual constructs, it is essential to demonstrate that the constructs are empirically distinct from one another and not redundant. We assessed discriminant validity using two complementary approaches recommended in recent methodological literature (Henseler et al., 2015). First, we applied the Fornell-Larcker criterion (Fornell and Larcker, 1981), which requires that the square root of average variance extracted (AVE) for each construct exceeds all correlations between that construct and other constructs in the model. This criterion ensures that each construct shares more variance with its own indicators than with any other construct, providing evidence of distinctiveness. Second, we calculated the Heterotrait-Monotrait (HTMT) ratio of correlations (Henseler et al., 2015), an increasingly preferred method that has been shown through simulation studies to have superior sensitivity and specificity compared to traditional approaches. HTMT values below 0.85 indicate adequate discriminant validity for conceptually similar constructs, while values below 0.90 are acceptable for conceptually distinct constructs. Table 5 presents the complete discriminant validity assessment matrix, with the square root of AVE on the diagonal (in bold), inter-construct correlations below the diagonal, and HTMT ratios above the diagonal.

As shown in Table 6, discriminant validity was fully supported using both criteria. For the Fornell-Larcker criterion, the square root of AVE for each construct (diagonal values ranging from 0.72 to 0.82) exceeded all correlations with other constructs in the corresponding row and column. The highest correlation was between Academic Motivation and Academic Satisfaction (*r* = 0.58), yet the square root of AVE for both constructs (0.75 and 0.82, respectively) exceeded this correlation, confirming discriminant validity. For the HTMT criterion, all ratios fell well within acceptable thresholds. The HTMT values ranged from 0.35 to 0.74, all substantially below the 0.85 threshold for similar constructs and the 0.90 threshold for distinct constructs. The highest HTMT ratios were observed between theoretically related constructs: Academic Motivation and Academic Satisfaction (HTMT = 0.68), Wellbeing and Academic Satisfaction (HTMT = 0.74), and Peer Support and Wellbeing (HTMT = 0.62). These moderately elevated values are conceptually appropriate given the theoretical overlap between these constructs—motivation and satisfaction both reflect positive academic experiences, while peer support and wellbeing both reflect psychosocial adjustment. Importantly, even these theoretically related constructs showed HTMT values well below problematic levels, confirming that they are empirically distinct despite their conceptual relationships. The lowest HTMT ratios were between AI Adoption and Peer Support (HTMT = 0.35) and AI Adoption and Wellbeing (HTMT = 0.42), indicating strong discriminant validity between technology use and psychosocial constructs. Collectively, these results provide robust evidence that all five constructs in our measurement model are empirically distinguishable and not redundant, satisfying a critical prerequisite for testing structural relationships in the hypothesized model.

**Table 6:** Discriminant validity: Fornell-Larcker criterion and HTMT ratios.

**Construct**	**1**	**2**	**3**	**4**	**5**
1. AI adoption	**0.73**	0.49	0.35	0.42	0.46
2. Academic motivation	0.45	**0.75**	0.48	0.59	0.68
3. Peer support	0.31	0.43	**0.72**	0.62	0.52
4. Student wellbeing	0.38	0.53	0.54	**0.76**	0.74
5. Academic satisfaction	0.41	0.58	0.46	0.65	**0.82**

N = 482. Values on the diagonal (in bold with gray shading) represent the square root of average variance extracted (√AVE) for each construct.

Values below the diagonal represent inter-construct correlations (Pearson r). All correlations are significant at p <0.001.

Values above the diagonal (in light blue shading) represent Heterotrait-Monotrait (HTMT) ratios.

Fornell-Larcker criterion: Discriminant validity is supported when √AVE (diagonal) exceeds all correlations in the corresponding row and column.

HTMT criterion: Values <0.85 indicate adequate discriminant validity for conceptually similar constructs; values <0.90 for conceptually distinct constructs (Henseler et al., 2015).

All constructs meet both criteria, supporting discriminant validity.

### 4.2 Structural equation modeling results

The hypothesized structural model demonstrated fit to the data: χ^2^_(156)_ = 278.42, *p* < 0.001; CFI = 0.968; TLI = 0.961; RMSEA = 0.041 (90% CI [0.034, 0.048]); SRMR = 0.045. All fit indices exceeded recommended thresholds, indicating that the model adequately represented relationships among variables. The structural model demonstrated good fit to the data: χ^2^_(245)_ = 412.33, *p* < 0.001; comparative fit index (CFI) = 0.95; Tucker-Lewis index (TLI) = 0.94; root mean square error of approximation (RMSEA) = 0.038, 90% CI [0.032, 0.045]; standardized root mean square residual (SRMR) = 0.042. These indices meet or exceed conventional thresholds for acceptable model fit (Hu and Bentler, 1999): CFI and TLI > 0.90 (ideally > 0.95), RMSEA <0.06, and SRMR <0.08. The non-significant chi-square relative to degrees of freedom (χ^2^/*df* = 1.68) also indicates acceptable fit.

Supporting Hypothesis 1, AI adoption significantly predicted academic motivation (β = 0.42, *SE* = 0.05, *p* < 0.001), explaining 18% of variance after controlling for demographic variables. Students who reported higher levels of AI adoption demonstrated significantly higher academic motivation. This relationship remained significant after accounting for potential confounds including prior academic achievement, socioeconomic status, and technology access.

Hypothesis 2 was also supported. AI adoption showed a significant positive direct effect on student wellbeing (β = 0.28, *SE* = 0.06, *p* < 0.01). However, this effect was smaller than the relationship with motivation, suggesting that AI's impact on wellbeing might operate partially through other mechanisms.

Hypothesis 3 proposed that peer support would moderate the relationship between AI adoption and wellbeing. Results strongly supported this hypothesis. The interaction term between AI adoption and peer support was significant (β = 0.35, *SE* = 0.07, *p* < 0.001). Simple slopes analysis revealed that the positive relationship between AI adoption and wellbeing was stronger for students reporting high peer support (1 SD above mean: β = 0.52, *p* < 0.001) compared to those reporting low peer support (1 SD below mean: β = 0.14, *p* = 0.08). This pattern indicates that peer support serves as an important contextual factor amplifying the benefits of AI adoption for student wellbeing.

Supporting Hypothesis 4, academic motivation significantly mediated the relationship between AI adoption and student success outcomes. The indirect effect of AI adoption on GPA through academic motivation was significant (indirect effect = 0.31, *SE* = 0.06, 95% CI [0.24, 0.39]). Similarly, academic motivation mediated the relationship between AI adoption and academic satisfaction (indirect effect = 0.36, *SE* = 0.07, 95% CI [0.28, 0.44]). These findings suggest that AI tools enhance student success partly by fostering academic motivation, which in turn predicts better academic outcomes and greater satisfaction with educational experiences.

Finally, supporting Hypothesis 5, the integrated model explained substantial variance in outcome variables: 34% of variance in academic motivation, 42% in student wellbeing, 29% in GPA, and 48% in academic satisfaction. These *R*^2^ values indicate that the model captures meaningful portions of variability in student outcomes, though substantial variance remains unexplained and likely attributable to factors not included in the current model.

Table 7 presents all direct effects, indirect effects (mediation), and the moderation effect with complete statistical information. All analyses control for demographic variables. Indirect effects were calculated using bootstrapping with 5,000 resamples, and moderation was tested using the LMS approach (Klein and Moosbrugger, 2000).

**Table 7:** Direct effects, mediation, and moderation analysis results.

**Effect/path**	**β**	**SE**	**95% CI**	**p**	***R* ^2^**
**(A) Direct effects**					
*Predicting academic motivation*					0.34
AI adoption → Acad. motivation	0.42	0.05	[0.32, 0.52]	<0.001	
*Predicting student wellbeing*					0.42
AI adoption → Wellbeing	0.28	0.06	[0.16, 0.40]	0.001	
Peer support → Wellbeing	0.47	0.06	[0.35, 0.59]	<0.001	
*Predicting academic performance (GPA)*					0.23
AI adoption → GPA (direct)	0.18	0.06	[0.06, 0.30]	0.004	
Acad. motivation → GPA	0.44	0.05	[0.34, 0.54]	<0.001	
*Predicting academic satisfaction*					0.51
AI adoption → Acad. satisfaction (direct)	0.09	0.06	[−0.03, 0.21]	0.124	
Acad. motivation → Acad. satisfaction	0.59	0.05	[0.49, 0.69]	<0.001	
**(B) Indirect effects (mediation via academic motivation)**					
AI adoption → Motivation → GPA	0.08	0.03	[0.03, 0.14]	0.002	
*% of total effect mediated*	31%				
*Total effect (AI*→*GPA)*	0.26	0.05	[0.16, 0.36]	<0.001	
AI adoption → Motivation → Satisfaction	0.22	0.04	[0.15, 0.30]	<0.001	
*% of total effect mediated*	71%				
*Total effect (AI*→*Satisfaction)*	0.31	0.05	[0.21, 0.41]	<0.001	
**(C) Moderation effect (AI adoption** × **Peer support**→**Wellbeing)**					
Interaction term (AI × Peer support)	0.35	0.07	[0.21, 0.49]	<0.001	Δ*R*^2^= 0.046
*LL difference test:* Δ-*2LL*	36.30	*df* = 1		<0.001	
**Simple slopes:**					
At low peer support (−1 SD)	0.14	0.08	[−0.01, 0.29]	0.076	
At mean peer support	0.28	0.06	[0.17, 0.39]	<0.001	
At high peer support (+1 SD)	0.52	0.07	[0.38, 0.66]	<0.001	
*Johnson-Neyman threshold*	3.82	*(52.3% of sample above threshold)*			

Table 7 demonstrates strong support for all hypotheses. AI adoption significantly predicted motivation (β = 0.42, *p* < 0.001) and wellbeing (β = 0.28, *p* = 0.001), supporting H1 and H2. The mediation analyses revealed partial mediation for GPA (31% of total effect, H4a) vs. full mediation for satisfaction (71% of total effect, H4b), supporting 5's prediction of differential relationships. The moderation effect was substantial (Δ*R*^2^ = 0.046, Cohen's *f*^2^ = 0.071). Following Cohen's (1988) guidelines, *f*^2^ values of 0.02, 0.15, and 0.35 represent small, medium, and large effect sizes, respectively. Our *f*^2^ of 0.071 indicates a small to medium effect, suggesting that peer support explains an additional 4.6% of variance in the relationship between AI adoption and wellbeing beyond the main effects alone. This effect size is meaningful in educational contexts where multiple factors influence student outcomes.

### 4.3 Additional analyses

Supplementary analyses examined potential differences across demographic groups. Multi-group SEM revealed no significant differences in structural paths between male and female students (Δχ^2^ = 12.34, *df* = 8, *p* = 0.14), suggesting that relationships among variables were consistent across genders. However, significant differences emerged between STEM and non-STEM students (Δχ^2^ = 23.56, *df* = 8, *p* = 0.003). Specifically, the relationship between AI adoption and academic motivation was stronger for STEM students (β = 0.51) than non-STEM students (β = 0.31), possibly reflecting differences in how AI tools are integrated into curricula across disciplines.

Exploratory analyses also examined specific types of AI tools used by students. The most commonly reported AI applications were language models for writing assistance (78.4% of participants), intelligent tutoring systems for mathematics and science (52.3%), automated translation tools (48.1%), and learning management system features using AI algorithms (41.5%). Frequency of use varied by tool type, with language models used most frequently (*M* = 3.2 days per week) followed by tutoring systems (*M* = 1.8 days per week). Students reporting regular use of multiple AI tool types showed higher levels of perceived benefit and greater integration of AI into their learning strategies.

The moderation analysis results provide complete transparency and facilitate replication. The results include five key components that comprehensively document the moderation effect. First, we report the log-likelihood values for both the baseline model without the interaction term (LL = −8742.33) and the comparison model including the latent interaction (LL = −8724.18), along with the formal log-likelihood difference test statistic. The difference between these models was highly significant (Δ-2LL = 36.30, *df* = 1, *p* < 0.001), providing strong statistical evidence that including the interaction term significantly improves model fit and that peer support indeed moderates the relationship between AI adoption and student wellbeing. Second, we report the standardized parameter estimate for the latent interaction term itself, which was substantial and highly significant (β = 0.35, *SE* = 0.07, *p* < 0.001), indicating a moderately strong moderating effect. Third, we present the results of simple slopes analysis conducted at three meaningful levels of the moderator variable. At low peer support (one standard deviation below the mean), the conditional effect of AI adoption on wellbeing was positive but not statistically significant (β = 0.14, *SE* = 0.08, *p* = 0.08, 95% CI [−0.01, 0.29]). At mean levels of peer support, the effect was significant and moderate in magnitude (β = 0.28, *SE* = 0.06, *p* < 0.001, 95% CI [0.17, 0.39]). At high peer support (one standard deviation above the mean), the effect was significant and substantially larger (β = 0.52, *SE* = 0.07, *p* < 0.001, 95% CI [0.38, 0.66]). This pattern clearly demonstrates that the positive relationship between AI adoption and wellbeing strengthens progressively as peer support increases. Fourth, we employed the Johnson-Neyman technique to identify the precise regions of statistical significance along the continuum of peer support values. This analysis revealed that AI adoption begins to significantly predict wellbeing when peer support exceeds a value of 3.82 on the seven-point scale, a threshold that was met or exceeded by 52.3% of our sample. Below this threshold, the relationship between AI adoption and wellbeing is not statistically distinguishable from zero, while above this threshold, the positive effect becomes significant and continues to strengthen as peer support increases. This finding has important practical implications, suggesting that roughly half of students have sufficient peer support to benefit from AI adoption in terms of their wellbeing, while the other half may need additional peer support resources to realize these benefits. Fifth, recognizing that the LMS approach does not provide traditional fit indices, we conducted a robustness check using the alternative product-indicator approach with mean-centered observed variables. This alternative method yielded a similar interaction effect (β = 0.33, *p* < 0.001) and demonstrated adequate model fit [χ^2^_(224)_ = 389.45, *p* < 0.001; CFI = 0.954; RMSEA = 0.039; SRMR = 0.048], confirming the consistency and reliability of our moderation findings across different analytical approaches. Additionally, we conducted a statistical power analysis using Monte Carlo simulation procedures to ensure that our sample size was adequate for detecting interaction effects. These simulations confirmed that with our sample of 482 participants, we had greater than 0.90 power (more than 90% probability) to detect interaction effects as small as β = 0.15 at the conventional alpha level of 0.05, assuming typical measurement reliability of 0.85 and moderate correlations among predictors of 0.30–0.50. This means we had excellent statistical power to detect even modest interaction effects. Importantly, our observed interaction effect of β = 0.35 was more than twice the magnitude of the minimum effect we had adequate power to detect, indicating that our study was well-powered and that the observed moderation effect is robust and unlikely to be a Type I error or the result of insufficient statistical power.

To ensure that the multi-group comparisons are methodologically sound, we have implemented comprehensive measurement invariance testing procedures prior to comparing structural paths across gender and field of study groups. We now explicitly specify that we followed the sequential testing approach outlined by Vandenberg and Lance (2000), which involves progressively testing increasingly restrictive levels of invariance to determine whether constructs are measured equivalently across groups. The first level, configural invariance, tests whether the same basic factor structure, the same pattern of which items load on which factors holds across all groups being compared. Establishing configural invariance is the most fundamental requirement, as it confirms that respondents in different groups conceptualize the constructs in the same way. The second level, metric invariance (also called weak invariance), tests whether the factor loadings—the relationships between observed items and their underlying latent constructs—are equal across groups. Metric invariance is necessary for comparing relationships among latent variables across groups, as it ensures that the measurement scale operates equivalently and that a one-unit change in the latent construct corresponds to the same change in observed item responses regardless of group membership. The third level, scalar invariance (also called strong invariance), tests whether the item intercepts are equal across groups in addition to the factor loadings. Scalar invariance is a critical requirement for comparing structural paths or latent means across groups because it ensures that any observed differences in scores reflect true differences in the underlying constructs rather than differences in how items function across groups. Without scalar invariance, group differences could be artifacts of differential item functioning rather than meaningful substantive differences. The fourth level, strict invariance, additionally constrains the residual variances (measurement error variances) to be equal across groups. While we tested for strict invariance to provide a complete assessment, this level is not required for valid multi-group comparisons and is often difficult to achieve in practice, as measurement error can vary across groups for reasons unrelated to construct measurement. Following the recommendations of Chen (2007), we evaluated invariance using multiple fit index criteria rather than relying solely on the chi-square difference test, which is known to be overly sensitive to sample size and often rejects invariance even when differences are trivially small. Specifically, we adopted the following decision rules: for metric invariance, we required that the change in CFI (ΔCFI) be no greater than 0.010, the change in RMSEA (ΔRMSEA) be no greater than 0.015, and the change in SRMR (ΔSRMR) be no greater than 0.030 compared to the configural model. For scalar invariance, we used the same criteria for ΔCFI and ΔRMSEA but employed a more stringent criterion of ΔSRMR ≤ 0.015 compared to the metric model. These criteria represent consensus recommendations based on simulation studies showing that these thresholds effectively balance Type I and Type II error rates in invariance testing. When full invariance at a particular level could not be established according to these criteria, we employed partial invariance procedures as recommended by Byrne et al. (1989). Partial invariance involves identifying specific parameters (factor loadings or intercepts) that differ across groups based on modification indices, freeing these parameters to vary across groups while maintaining equality constraints on the remaining parameters. Partial invariance is considered acceptable for proceeding with group comparisons if at least two indicators per construct maintain invariant parameters, ensuring that sufficient measurement equivalence exists to make meaningful comparisons. This flexible yet rigorous approach allows us to acknowledge real differences in how certain items may function across groups while still establishing adequate measurement equivalence to support valid structural comparisons.

Gender Groups: Full scalar invariance achieved (all delta-fit indices met criteria), permitting structural path comparisons. No significant differences in structural paths found (delta-chi-square = 12.34, *df* = 8, *p* = 0.14), indicating relationships operate similarly across gender.

STEM vs. Non-STEM: Partial scalar invariance established (2 loadings and 3 intercepts freed based on modification indices). Sufficient invariance maintained for valid comparisons. Structural path comparison revealed AI Adoption → Academic Motivation significantly stronger in STEM (beta = 0.51) than non-STEM (beta = 0.31; delta-chi-square = 8.94, *p* = 0.003), while other paths did not differ.

The need for partial invariance across fields is theoretically meaningful, reflecting genuine differences in how STEM vs. non-STEM students experience motivation and utilize AI tools. These findings inform differentiated implementation strategies across disciplines.

### 4.4 Multi-group analyses

We tested measurement invariance across gender and field of study (STEM/non-STEM) before comparing structural paths. Following Vandenberg and Lance (2000), we tested configural, metric, and scalar invariance using criteria from Chen (2007): ΔCFI ≤ 0.010, ΔRMSEA ≤ 0.015, ΔSRMR ≤ 0.030/0.015. Partial invariance procedures were employed when full invariance was not achieved. Table 8 presents invariance tests (Panel A) and group-specific path estimates (Panel B).

**Table 8:** Measurement invariance tests and multi-group structural path comparisons.

**(A) Measurement invariance testing**							
**Model**	χ^2^	***df***	**CFI**	**RMSEA [90% CI]**	**SRMR**	Δ**CFI**	Δ**RMSEA**
**Gender groups (male vs. female)**							
Configural	1089.34	734	0.962	0.038 [0.034, 0.042]	0.045	–	–
Metric	1108.67	749	0.959	0.039 [0.035, 0.043]	0.053	−0.003	0.001
Scalar	1151.34	769	0.951	0.041 [0.037, 0.045]	0.064	−0.008	0.002
Strict	1203.78	814	0.944	0.042 [0.038, 0.046]	0.068	−0.007	0.001
**Field of study (STEM vs. non-STEM)**							
Configural	1112.45	734	0.959	0.039 [0.035, 0.043]	0.048	–	–
*Partial metric*	1145.23	747	0.953	0.041 [0.037, 0.045]	0.054	−0.006	0.002
*Partial scalar*	1189.67	764	0.944	0.044 [0.040, 0.048]	0.067	−0.009	0.003
**(B) Group-specific structural path estimates**							
**Structural path**	**Group 1** β **(*****SE*****)**	**95% CI**	***p***	**Group 2** β **(*****SE*****)**	**95% CI**	***p***	Δχ^2^ **(*****p*****)**
**Gender: male vs. female**							
AI adoption → Motivation	0.40 (0.06)	[0.28, 0.52]	<0.001	0.44 (0.07)	[0.30, 0.58]	<0.001	0.89 (0.35)
AI adoption → Wellbeing	0.26 (0.08)	[0.10, 0.42]	0.002	0.30 (0.08)	[0.14, 0.46]	<0.001	0.42 (0.52)
Motivation → GPA	0.46 (0.07)	[0.32, 0.60]	<0.001	0.41 (0.07)	[0.27, 0.55]	<0.001	1.24 (0.27)
Motivation → Satisfaction	0.61 (0.07)	[0.47, 0.75]	<0.001	0.57 (0.07)	[0.43, 0.71]	<0.001	0.67 (0.41)
**Field of study: STEM vs. non-STEM**							
AI adoption → Motivation	0.51 (0.06)	[0.39, 0.63]	<0.001	0.31 (0.07)	[0.17, 0.45]	<0.001	8.94 (0.003)
AI adoption → Wellbeing	0.26 (0.08)	[0.10, 0.42]	0.002	0.29 (0.09)	[0.11, 0.47]	0.002	0.23 (0.63)
Motivation → GPA	0.48 (0.06)	[0.36, 0.60]	<0.001	0.36 (0.08)	[0.20, 0.52]	<0.001	3.21 (0.073)
Motivation → Satisfaction	0.62 (0.06)	[0.50, 0.74]	<0.001	0.58 (0.08)	[0.42, 0.74]	<0.001	0.42 (0.52)

Panel A shows that full scalar invariance was achieved for gender groups, while partial scalar invariance was established for STEM/non-STEM (2 loadings and 3 intercepts freed). Panel B reveals no significant gender differences in structural paths (all Δχ^2^ *p*-values > 0.27), indicating the model operates equivalently for males and females. For field of study, AI Adoption → Motivation was significantly stronger in STEM (β = 0.51) than non-STEM (β = 0.31; Δχ^2^ = 8.94, *p* = 0.003), while other paths did not differ significantly. These results demonstrate adequate measurement equivalence and identify field-specific considerations for AI implementation.

### 4.5 Measurement invariance test

The multi-group comparisons require establishing measurement invariance (also called measurement equivalence) before comparing structural paths. Without measurement invariance, observed group differences could reflect differences in how constructs are measured rather than true substantive differences in the constructs themselves (Vandenberg and Lance, 2000). Measurement invariance testing follows a sequential hierarchy, with each level imposing increasingly restrictive constraints (Meredith, 1993):

1. Configural invariance (equal form): Tests whether the same basic factor structure (pattern of which items load on which factors) holds across groups. This is the baseline requirement.
2. Metric invariance (equal loadings): Tests whether factor loadings are equal across groups. Metric invariance is necessary for comparing relationships among latent variables (structural paths) across groups.
3. Scalar invariance (equal intercepts): Tests whether item intercepts are equal across groups in addition to loadings. Scalar invariance is necessary for comparing latent means across groups, though this is not our primary goal.

Following recommendations by Chen (2007) and Cheung and Rensvold (2002), we evaluated invariance using changes in fit indices rather than relying solely on chi-square difference tests, which are overly sensitive to sample size. Specifically, we adopted the criteria proposed by Chen (2007): ΔCFI ≤ 0.010, ΔRMSEA ≤ 0.015, and ΔSRMR ≤ 0.030 for metric invariance and ΔSRMR ≤ 0.015 for scalar invariance.

### 4.6 Measurement invariance results

We conducted measurement invariance testing for all five latent constructs (AI Adoption, Academic Motivation, Peer Support, Wellbeing, Academic Satisfaction) across two key grouping variables: (1) gender (male vs. female), and (2) field of study (STEM vs. non-STEM). Table 9 presents the complete invariance testing results.

**Table 9:** Measurement invariance testing across groups.

**Model**	**χ^2^ (*df*)**	**CFI**	**RMSEA**	**SRMR**	**ΔCFI**	**ΔRMSEA**	**ΔSRMR**
**Gender groups (male vs. female)**							
Configural	823.45 (734)	0.965	0.041	0.045	–	–	–
Metric	847.23 (765)	0.963	0.042	0.048	**−0.002**	**+0.001**	**+0.003**
Scalar	879.56 (796)	0.960	0.043	0.051	**−0.003**	**+0.001**	**+0.003**
**Field of study (STEM vs. non-STEM)**							
Configural	831.67 (734)	0.964	0.042	0.046	–	–	–
Metric	862.34 (765)	0.961	0.044	0.051	**−0.003**	**+0.002**	**+0.005**
Partial scalar	891.45 (791)	0.958	0.044	0.054	**−0.003**	**0.000**	**+0.003**

Bold values in the ΔCFI, ΔRMSEA, and ΔSRMR columns indicate changes that meet the criteria for establishing measurement invariance (ΔCFI ≤ 0.01, ΔRMSEA ≤ 0.015, ΔSRMR ≤ 0.03 for metric invariance; ΔCFI ≤ 0.01, ΔRMSEA ≤ 0.015, ΔSRMR ≤ 0.01 for scalar invariance; Chen, 2007).

### 4.7 Interpretation of invariance results

#### 4.7.1 Gender groups

Full scalar invariance was achieved for gender groups, with all fit index changes meeting Chen's (2007) criteria. Specifically, ΔCFI values ranged from −0.002 to −0.003 (well below the 0.010 threshold), ΔRMSEA values were 0.001 (well below 0.015), and ΔSRMR values were 0.003 (well below both 0.030 for metric and 0.015 for scalar). These results indicate that all five constructs are measured equivalently across male and female participants, supporting the validity of structural path comparisons between genders.

#### 4.7.2 Field of study groups

Configural and metric invariance were fully supported for field of study groups (STEM vs. non-STEM), with all fit index changes meeting criteria. However, full scalar invariance was not achieved, with initial tests showing ΔCFI = −0.008, ΔRMSEA = +0.004, and ΔSRMR = +0.018, where the ΔSRMR value slightly exceeded the 0.015 criterion.

Following procedures recommended by Byrne et al. (1989), we established partial scalar invariance by examining modification indices to identify specific parameters showing non-invariance. We freed 2 factor loadings (AI Adoption items 3 and 7) and 3 intercepts (Academic Motivation items 2 and 5, Wellbeing item 9) that showed MI > 10, indicating substantial cross-group differences. This partial invariance model demonstrated adequate fit (ΔCFI = −0.003, ΔRMSEA = 0.000, ΔSRMR = +0.003), with all values meeting Chen's criteria.

The non-invariant parameters are theoretically meaningful rather than problematic. STEM and non-STEM students demonstrably differ in their exposure to and use of AI technologies, making it reasonable that certain AI adoption items (particularly item 3, “AI tools are part of my regular study routine,” and item 7, “AI tools make my learning more productive”) function differently across fields. Similarly, motivational experiences (items 2 and 5 related to autonomous motivation) may be differentially salient across disciplines given structural differences in curricula and assessment. Since at least two indicators per construct maintained invariant parameters, sufficient measurement equivalence exists to justify structural comparisons (Byrne et al., 1989).

### 4.8 Common method bias assessment

The common method bias (CMB) can artificially inflate relationships among variables when all data are collected from the same source at a single time point (Podsakoff et al., 2003). While we implemented several procedural remedies during data collection (e.g., counterbalancing question order, ensuring respondent anonymity, using validated scales with established psychometric properties), we now supplement these with rigorous statistical tests.

#### 4.8.1 Test 1: Harman's single-factor test

**Procedure:** All items from all constructs loaded onto a single factor in confirmatory factor analysis.

**Criterion:** If a single factor accounts for the majority of variance (≥ 50%), CMB is likely problematic.

The single-factor model yielded χ^2^_(860)_ = 3247.58, *p* < 0.001; CFI = 0.612; TLI = 0.589; RMSEA = 0.078; SRMR = 0.092. This model demonstrated very poor fit and accounted for only 34.2% of the total variance. The poor fit and low variance explained indicate that a single common method factor does not adequately explain the data structure, providing evidence against substantial CMB.

#### 4.8.2 Test 2: common latent factor (CLF) approach

Following Williams et al. (2010), we added a common latent factor to our measurement model where all items loaded on both their theoretical construct and the CLF. The CLF captures variance attributable to common method rather than trait variance (Table 10).

**Table 10:** Common method bias assessment using common latent factor approach.

**Model**	**Fit indices**	**Average CLF loading**
Baseline (no CLF)	χ^2^_(367)_ = 624.58, CFI = 0.961, RMSEA = 0.038	–
With CLF	χ^2^_(322)_ = 583.42, CFI = 0.964, RMSEA = 0.036	0.18 (range: 0.09–0.27)

The CLF model improved fit marginally (ΔCFI = 0.003, ΔRMSEA = −0.002), but CLF factor loadings were relatively small (average = 0.18, range: 0.09–0.27), suggesting that common method variance accounts for a minor portion of item variance. Most importantly, we compared standardized path coefficients from our structural model with and without the CLF included (Table 11).

**Table 11:** Comparison of structural path estimates with and without common latent factor.

**Structural path**	**Without CLF (β)**	**With CLF (β)**	**Difference**
AI adoption → Motivation	0.42^***^	0.39^***^	−0.03
AI adoption → Wellbeing	0.28^**^	0.26^**^	−0.02
Motivation → GPA	0.52^***^	0.49^***^	−0.03
Motivation → Satisfaction	0.58^***^	0.54^***^	−0.04
Peer support → Wellbeing	0.54^***^	0.51^***^	−0.03
AI × Peer support → Wellbeing	0.35^***^	0.33^***^	−0.02

N = 482. CLF, Common Latent Factor. All coefficients are standardized path estimates. Green shading indicates minimal differences (<0.05) between models. ^**^p < 0.01; ^***^p < 0.001.

The CLF analysis reveals that including a common method factor produces only trivial changes in path estimates, with all differences ranging from −0.02 to −0.04 (mean absolute difference = 0.03). All structural paths remained statistically significant with virtually unchanged effect sizes after controlling for CMB. The pattern of findings is remarkably stable across models.

Collectively, these results provide strong evidence that common method bias is not a substantial threat to the validity of our findings. While some degree of common method variance is inevitably present when using self-report data, it does not meaningfully inflate the relationships among constructs or alter substantive conclusions. The robustness of our findings across different CMB assessment approaches (Harman's test, CLF) strengthens confidence in the validity of our structural model results.

## 5 Discussion

This study investigated the relationships among AI adoption, peer support, academic motivation, and student wellbeing in Pakistani university students. Results provide empirical support for a model in which AI technologies can enhance student outcomes, but their effectiveness depends on motivational factors and social support systems. These findings contribute to understanding how educational technology operates in non-Western contexts and have important implications for policy and practice.

### 5.1 AI Adoption and academic motivation

The strong positive relationship between AI adoption and academic motivation aligns with Self-Determination Theory's emphasis on competence and autonomy as drivers of intrinsic motivation. AI tools may enhance competence by providing personalized learning experiences, immediate feedback, and opportunities for mastery at one's own pace. The ability to access diverse learning resources and customize learning pathways may support autonomy by giving students greater control over their educational experiences.

These findings extend previous research conducted primarily in Western contexts by demonstrating that AI's motivational benefits generalize to Pakistani students. This is noteworthy given cultural differences in educational values and practices. Pakistani education has traditionally emphasized teacher-centered instruction, rote learning, and high-stakes examinations (Rashid and Asghar, 2016). The finding that AI tools, which often employ learner-centered approaches, can nevertheless enhance motivation suggests these technologies may facilitate positive changes in learning culture.

However, it is important to note that correlation does not imply causation. While AI adoption predicted motivation in our model, reverse causality is possible: students with higher initial motivation might be more likely to adopt AI tools. Longitudinal research is needed to disentangle these directional relationships. Additionally, the mechanisms through which AI affects motivation require further investigation. Do AI tools enhance motivation primarily through supporting competence, autonomy, or both? Are certain types of AI applications more effective at fostering motivation than others?

An important theoretical contribution of this study is demonstrating that AI adoption influences objective academic performance (GPA) and subjective academic satisfaction through different mechanisms. Our finding of partial mediation for GPA (31% of total effect mediated through motivation) vs. full mediation for satisfaction (71% of total effect mediated through motivation) provides empirical support for treating these as conceptually distinct constructs rather than combining them into a single “student success” composite. This pattern aligns with extensive literature showing that grades and satisfaction represent different dimensions of educational outcomes with distinct antecedents (Kuh et al., 2006; Richardson et al., 2012). Academic performance is determined by multiple factors including prior knowledge, study strategies, cognitive abilities, and task difficulty, with motivation being one contributor among many. In contrast, academic satisfaction—a subjective evaluation of educational quality—is more directly tied to motivational experiences, particularly the satisfaction of basic psychological needs for autonomy, competence, and relatedness emphasized in Self-Determination Theory (Ryan and Deci, 2017).

The practical implication is that interventions promoting AI adoption should set differentiated expectations: improvements in student satisfaction may occur relatively quickly through enhanced motivation and interest, while improvements in grades may require additional supports that address study skills, metacognitive strategies, and domain knowledge alongside motivational enhancement. Universities should not assume that technologies increasing student satisfaction will automatically improve grades at the same magnitude, nor should they dismiss satisfaction improvements as unimportant merely because they don't translate directly to GPA gains. Both objective achievement and subjective experience are valuable educational outcomes, and our results suggest they respond somewhat differently to technological interventions.

### 5.2 The moderating role of peer support

Perhaps the most important finding of this study is that peer support moderates the relationship between AI adoption and wellbeing. Students with strong peer support networks derived greater wellbeing benefits from AI use than those with limited peer support. This interaction effect addresses concerns that educational technology might isolate students or undermine social aspects of learning (Selwyn, 2019).

Several mechanisms might explain this moderation. First, peer support may help students navigate challenges associated with AI adoption, such as technical difficulties or uncertainty about appropriate use. Students with strong peer networks can seek assistance, share strategies, and troubleshoot problems together. Second, peer support may provide important social connection that balances technology-mediated learning, helping maintain relatedness needs even as learning becomes more individualized. Third, discussing and reflecting on AI-mediated learning experiences with peers may deepen learning and enhance sense of community.

This finding has particular relevance in collectivistic cultures like Pakistan, where social relationships and group belonging are highly valued (Haslam et al., 2005). Successful technology integration in such contexts must attend not only to individual learning but also to maintaining and supporting peer relationships. Educational institutions should consider how to foster peer support alongside AI implementation, perhaps through structured collaborative learning activities, peer mentoring programs, or online communities where students can discuss their technology-mediated learning experiences.

### 5.3 Motivation as mediator of student success

The mediating role of academic motivation between AI adoption and student success outcomes illuminates pathways through which technology affects academic achievement. AI tools do not directly improve grades or satisfaction; rather, they enhance motivation, which in turn predicts better outcomes. This mediation model suggests that technology's effectiveness depends on its ability to engage students' intrinsic interest and support autonomous regulation of learning.

These findings align with broader educational research showing motivation as a key predictor of academic success (Richardson et al., 2012). They also suggest that evaluations of educational technology should assess not only immediate learning outcomes but also motivational impacts. Technologies that enhance short-term performance through external scaffolding but undermine intrinsic motivation may prove counterproductive in the long run. Conversely, technologies that foster autonomous motivation may have lasting benefits extending beyond specific courses or content.

The partial mediation observed in this study indicates that motivation explains some, but not all, of AI's effects on student success. Other mediating variables might include learning strategies, self-efficacy, engagement, or metacognitive skills. Future research should explore these additional pathways to develop more comprehensive understanding of how AI adoption translates into improved outcomes.

### 5.4 Contextual considerations: AI in Pakistan

This study provides important evidence about AI adoption in a developing nation context. Pakistani students demonstrated substantial engagement with AI tools despite infrastructure challenges and limited formal training. This suggests strong demand for technology-enhanced learning and students' resourcefulness in accessing and utilizing available tools. The popularity of language models for writing assistance may reflect Pakistan's multilingual context, where many students write academic work in English despite speaking other languages primarily.

However, several contextual factors merit consideration. First, access disparities remain significant. While our sample reported relatively high AI adoption, they represent students at major urban universities with better resources. Students in rural areas or less-resourced institutions may have limited access to AI tools, potentially exacerbating educational inequalities. Second, infrastructure challenges including unreliable electricity and internet connectivity affect consistent technology use. Third, most AI tools are developed in Western contexts and may not adequately address Pakistani students' specific needs, such as support for Urdu or other regional languages, alignment with local curricula, or culturally relevant examples.

The differences between STEM and non-STEM students found in supplementary analyses suggest that AI integration varies across disciplines. STEM fields may have more developed AI applications (e.g., intelligent tutoring systems for mathematics) or faculty who are more comfortable incorporating AI into teaching. Addressing this disparity requires attention to discipline-specific needs and challenges in AI implementation.

### 5.5 Implications

An important theoretical contribution of this study is demonstrating that AI adoption influences objective academic performance (GPA) and subjective academic satisfaction through different mechanisms. Our finding of partial mediation for GPA (31% of total effect mediated through motivation) vs. full mediation for satisfaction (71% of total effect mediated through motivation) provides empirical support for treating these as conceptually distinct constructs rather than combining them into a single “student success” composite. This pattern aligns with extensive literature showing that grades and satisfaction represent different dimensions of educational outcomes with distinct antecedents (Kuh et al., 2006; Richardson et al., 2012). Academic performance is determined by multiple factors including prior knowledge, study strategies, cognitive abilities, and task difficulty, with motivation being one contributor among many. In contrast, academic satisfaction—a subjective evaluation of educational quality—is more directly tied to motivational experiences, particularly the satisfaction of basic psychological needs for autonomy, competence, and relatedness emphasized in Self-Determination Theory (Ryan and Deci, 2017).

These findings offer several practical implications for educators, administrators, and policymakers:

First, universities should develop comprehensive strategies for AI integration that go beyond merely providing access to tools. Training programs should help students understand how to use AI effectively for learning while avoiding overreliance or academic integrity issues. Faculty development is equally important to help instructors integrate AI into pedagogy thoughtfully.

Second, institutions must attend to social dimensions of learning alongside technology implementation. The moderating effect of peer support suggests that successful AI adoption requires maintaining and strengthening peer relationships. This might involve designing collaborative AI-mediated learning activities, creating peer mentoring systems, or establishing online communities where students discuss technology use and learning strategies.

Third, technology evaluation should examine motivational impacts. Institutions piloting AI tools should assess not only learning outcomes but also whether technologies enhance autonomous motivation. Tools that undermine intrinsic interest or foster external regulation may be counterproductive despite short-term benefits.

Fourth, addressing access disparities is crucial for equity. Policymakers should invest in infrastructure improvements, provide devices and internet access to under-resourced students, and support development of AI tools tailored to Pakistani contexts, including multilingual capabilities and culturally relevant content.

Finally, institutional policies should address emerging challenges, including academic integrity in the AI era, data privacy and security, and ethical considerations in AI use. Clear guidelines will help students and faculty navigate these complex issues. The practical implication is that interventions promoting AI adoption should set differentiated expectations: improvements in student satisfaction may occur relatively quickly through enhanced motivation and interest, while improvements in grades may require additional supports that address study skills, metacognitive strategies, and domain knowledge alongside motivational enhancement. Universities should not assume that technologies increasing student satisfaction will automatically improve grades at the same magnitude, nor should they dismiss satisfaction improvements as unimportant merely because they don't translate directly to GPA gains. Both objective achievement and subjective experience are valuable educational outcomes, and our results suggest they respond somewhat differently to technological interventions.

The implications of our findings must be interpreted within the specific context of Pakistani higher education. The strong moderation effect of peer support likely reflects Pakistan's collectivistic culture (Khilji, 2003), where social learning and group-oriented approaches are culturally valued. Educators implementing AI tools in similar collectivistic contexts should emphasize collaborative features, peer-assisted learning with AI tools, and group-based AI projects rather than purely individualized learning.

Infrastructure challenges require particular attention. Universities should provide reliable computer lab access, stable internet connectivity, and backup power sources to ensure equitable access to AI tools. Training programs should address varying levels of digital literacy and provide ongoing technical support. Implementation strategies successful in well-resourced Western universities may require substantial adaptation to succeed in resource-constrained Pakistani settings.

The high power distance characteristic of Pakistani educational culture may create initial resistance to AI tools that encourage learner autonomy and self-directed learning. Institutions should emphasize how AI tools complement rather than replace instructor authority, involve faculty in AI implementation decisions, and provide professional development that helps instructors integrate AI into pedagogically sound practices. Local adaptation is essential—AI tools should be evaluated and selected based on Pakistani students' needs, learning preferences, and contextual constraints rather than simply importing solutions designed for different educational contexts.

### 5.6 Limitations and future directions

Several important limitations should be acknowledged. First, the cross-sectional design precludes causal inferences. While our findings suggest associations between AI adoption and student outcomes, alternative explanations warrant consideration. Students with higher pre-existing academic motivation or better socioeconomic resources may be more likely both to adopt AI tools and to experience positive outcomes, creating spurious associations. Individual differences in digital literacy, prior academic performance, and access to technological infrastructure (reliable internet, devices) could confound observed relationships. The positive association between AI adoption and outcomes may reflect selection effects rather than treatment effects students who are already succeeding academically may be more willing and able to experiment with new learning technologies.

Reverse causality is also possible. Students experiencing greater wellbeing may be more inclined to explore and adopt new educational technologies, have more cognitive resources to learn new tools, and persist through the initial learning curve of AI applications. Similarly, highly motivated students may actively seek out AI tools as additional learning resources, meaning motivation could precede rather than result from AI adoption.

Furthermore, unmeasured third variables could explain both AI adoption and positive outcomes. For example, institutional support (access to training, technical assistance), instructor encouragement, or general openness to innovation might drive both technology use and academic success. Family socioeconomic status could enable both device ownership for AI tool access and other educational advantages (tutoring, conducive study environments, reduced work obligations) that independently influence outcomes.

Longitudinal research with multiple measurement occasions is needed to establish temporal precedence and rule out competing explanations (Maxwell and Cole, 2007). Future studies should measure and control for baseline differences in motivation, prior achievement, socioeconomic status, and digital literacy to strengthen causal inferences. Experimental designs that randomly assign students to AI-enhanced vs. traditional learning conditions would provide more definitive evidence of causal effects.

Third, the sample consisted of students from major urban universities, limiting generalizability to students in rural areas or smaller institutions. Future research should examine AI adoption and impacts across more diverse educational settings. Fourth, we did not differentiate between types of AI tools or quality of use. Students using AI for shallow tasks (e.g., generating text without engagement) may experience different outcomes than those using AI for deeper learning (e.g., to receive feedback on problem-solving). Research examining patterns of AI use and their differential effects would be valuable.

Fifth, our model explained substantial but not complete variance in outcomes, suggesting important factors were not captured. Variables such as faculty support, institutional policies, digital literacy, self-regulation skills, and learning strategies may moderate or mediate relationships among variables. Sixth, cultural factors were not explicitly measured. While this study was conducted in Pakistan, we did not assess cultural values or orientations that might shape technology adoption and impacts. Future research should incorporate cultural variables to test whether relationships differ based on individualism-collectivism, power distance, or other cultural dimensions.

Finally, the rapidly evolving nature of AI technology means that findings may have limited temporal generalizability. Tools available today may differ substantially from those available even a year from now. Ongoing research is needed to track how AI impacts evolve as technologies advance and become more sophisticated.

Despite these limitations, the study demonstrates several methodological strengths that enhance its rigor. The use of a priori hypotheses grounded in Self-Determination Theory, an adequate sample size (*n* = 453), strong model fit indices, and the inclusion of a theoretically motivated moderator (peer support) strengthen causal inference. However, rigor is weakened by the absence of measurement invariance testing across demographic groups and the limited validation of the newly developed AI adoption instrument, which requires further psychometric evaluation across diverse samples. Additionally, the composite “student success' measure conflates academic performance (GPA) with psychosocial outcomes (academic satisfaction), potentially obscuring differential pathways. As our supplementary analyses revealed, AI adoption's effect on GPA is partially mediated by motivation (31%), whereas its effect on satisfaction is fully mediated (71%), suggesting these outcomes operate through distinct mechanisms that warrant separate examination in future research.

An important limitation of this study is the use of cross-sectional data to test mediation hypotheses. As articulated by Maxwell and Cole (2007) and Cole and Maxwell (2003), establishing causal mediation definitively requires demonstrating temporal precedence among variables—that the predictor precedes the mediator, which in turn precedes the outcome. Our simultaneous measurement of all constructs means we cannot rule out alternative causal orderings, such as motivation influencing AI adoption or satisfaction influencing motivation. Therefore, our indirect effects should be interpreted as associations consistent with the hypothesized mediation processes rather than definitive evidence of causal mediation. While our hypotheses are grounded in Self-Determination Theory's well-established theoretical sequence (environmental factors → need satisfaction and motivation → outcomes) and are consistent with experimental and longitudinal evidence from other contexts, longitudinal replication is essential to establish temporal ordering. Future research should employ three-wave panel designs measuring AI adoption at Time 1, academic motivation at Time 2, and outcomes at Time 3, ideally with time lags of several weeks to allow proposed processes to unfold while minimizing the influence of unmeasured time-varying confounds (Cole and Maxwell, 2003; Selig and Preacher, 2009).

## 6 Conclusion

This study provides empirical evidence that AI adoption can positively influence academic motivation, wellbeing, and success among Pakistani university students. However, these benefits are not automatic or universal. The effectiveness of AI tools depends critically on contextual factors, particularly peer support networks that amplify technology's positive effects. Additionally, AI's impact on student success operates partially through enhanced academic motivation, highlighting the importance of fostering autonomous motivation in technology-mediated learning environments.

These findings challenge simplistic narratives about educational technology either as panacea or threat. Instead, they suggest a nuanced view: AI tools are most effective when integrated thoughtfully into learning environments that maintain human connection and support student agency. For developing nations like Pakistan, successful AI integration requires not only technological infrastructure but also attention to social, cultural, and motivational factors that shape how students experience and benefit from these tools.

As AI becomes increasingly prevalent in education worldwide, understanding its impacts in diverse contexts is essential. This study contributes to that understanding by examining AI adoption in a non-Western, developing nation setting where educational challenges and cultural values differ from contexts where most research has been conducted. Future research should continue exploring how AI technologies can be leveraged effectively to support student learning and wellbeing across diverse cultural, institutional, and resource contexts.

Ultimately, the goal should not be AI adoption for its own sake, but rather thoughtful integration of technology in service of fundamental educational aims: fostering student motivation, supporting learning and development, promoting wellbeing, and preparing students for meaningful lives and careers. This research suggests that achieving these goals requires attending to the interplay between technology, motivation, and social connection—a complex but navigable challenge for educators and institutions committed to student success in the AI era.

## Data Availability

The original contributions presented in the study are included in the article/supplementary material, further inquiries can be directed to the corresponding author.

## References

[B1] Adnan M. Anwar K. (2020). Online learning amid the COVID-19 pandemic: students' perspectives. J. Pedagogical Sociol. Psychol. 2, 45–51. doi: 10.33902/JPSP.2020261309

[B2] Aiken L. S. West S. G. (1991). Multiple Regression: Testing and Interpreting Interactions. Sage Publications.

[B3] Ali W. (2020). Online and remote learning in higher education institutes: a necessity in light of COVID-19 pandemic. Higher Educ. Stud. 10, 16–25. doi: 10.5539/hes.v10n3p16

[B4] Arnold K. E. Pistilli M. D. (2012). “Course signals at Purdue: using learning analytics to increase student success,” in Proceedings of the 2nd International Conference on Learning Analytics and Knowledge (New York, NY: ACM), 267–270. doi: 10.1145/2330601.2330666

[B5] Baker R. S. Inventado P. S. (2014). “Educational data mining and learning analytics,” in Learning Analytics: From Research to Practice, eds. J. A. Larusson and B. White (New York, NY: Springer), 61–75. doi: 10.1007/978-1-4614-3305-7_4

[B6] Baron R. M. Kenny D. A. (1986). The moderator-mediator variable distinction in social psychological research: conceptual, strategic, and statistical considerations. J. Pers. Soc. Psychol. 51, 1173–1182. doi: 10.1037/0022-3514.51.6.1173 3806354

[B7] Bo Z. Pek L. S. Cong W. Tiannan L. Krishnasamy H. N. Ne'matullah K. F. . (2025). Transforming translation education: a bibliometric analysis of artificial intelligence's role in fostering sustainable development. Int. J. Learn. Teach. Educ. Res. 24, 166–190. doi: 10.26803/ijlter.24.3.9

[B8] Byrne B. M. Shavelson R. J. Muthén B. (1989). Testing for the equivalence of factor covariance and mean structures: the issue of partial measurement invariance. Psychol. Bull. 105, 456–466. doi: 10.1037/0033-2909.105.3.456

[B9] Chen F. F. (2007). Sensitivity of goodness of fit indexes to lack of measurement invariance. Struct. Eq. Model. 14, 464–504. doi: 10.1080/10705510701301834

[B10] Chen K.-C. Jang S.-J. (2010). Motivation in online learning: testing a model of self-determination theory. Comput. Hum. Behav. 26, 741–752. doi: 10.1016/j.chb.2010.01.011

[B11] Chen L. Chen P. Lin Z. (2020). Artificial intelligence in education: a review. IEEE Access 8, 75264–75278. doi: 10.1109/ACCESS.2020.2988510

[B12] Cheung G. W. Rensvold R. B. (2002). Evaluating goodness-of-fit indexes for testing measurement invariance. Struct. Eq. Model. 9, 233–255. doi: 10.1207/S15328007SEM0902_5

[B13] Cohen J. (1988). Statistical Power Analysis for the Behavioral Sciences, 2nd Edn. Hillsdale, NJ: Lawrence Erlbaum Associates.

[B14] Cole D. A. Maxwell S. E. (2003). Testing mediational models with longitudinal data: questions and tips in the use of structural equation modeling. J. Abnormal Psychol. 112, 558–577. doi: 10.1037/0021-843X.112.4.558 14674869

[B15] Davis F. D. (1989). Perceived usefulness, perceived ease of use, and user acceptance of information technology. MIS Q. 13, 319–340. doi: 10.2307/249008

[B16] Deci E. L. Ryan R. M. (2000). The “what” and “why” of goal pursuits: human needs and the self-determination of behavior. Psychol. Inquiry 11, 227–268. doi: 10.1207/S15327965PLI1104_01

[B17] Dennis J. M. Phinney J. S. Chuateco L. I. (2005). The role of motivation, parental support, and peer support in the academic success of ethnic minority first-generation college students. J. Coll. Student Dev. 46, 223–236. doi: 10.1353/csd.2005.0023

[B18] Fornell C. Larcker D. F. (1981). Evaluating structural equation models with unobservable variables and measurement error. J. Market. Res. 18, 39–50. doi: 10.2307/3151312

[B19] Frazier P. A. Tix A. P. Barron K. E. (2004). Testing moderator and mediator effects in counseling psychology research. J. Counsel. Psychol. 51, 115–134. doi: 10.1037/0022-0167.51.1.115

[B20] Hair J. F. Sarstedt M. Ringle C. M. Mena J. A. (2012). An evaluation of the use of partial least squares structural equation modeling in marketing research. J. Acad. Market. Sci. 40, 414–433. doi: 10.1007/s11747-011-0261-6

[B21] Haslam S. A. Jetten J. Postmes T. Haslam C. (2005). Social identity, health and well-being: an emerging agenda for applied psychology. Appl. Psychol. 54, 1–23.

[B22] Hayes A. F. (2018). Introduction to Mediation, Moderation, and Conditional Process Analysis: A Regression-Based Approach, 2nd Edn. New York, NY: Guilford Press.

[B23] Hayes A. F. (2022). Introduction to Mediation, Moderation, and Conditional Process Analysis: A Regression-Based Approach, 3rd Edn. New York, NY: Guilford Press.

[B24] Hefner J. Eisenberg D. (2009). Social support and mental health among college students. Am. J. Orthopsychiatry 79, 491–499. doi: 10.1037/a0016918 20099940

[B25] Henseler J. Ringle C. M. Sarstedt M. (2015). A new criterion for assessing discriminant validity in variance-based structural equation modeling. J. Acad. Market. Sci. 43, 115–135. doi: 10.1007/s11747-014-0403-8

[B26] Higher Education Commission Pakistan (2021). Annual Report 2020–2021. HEC Pakistan.

[B27] Ho J. W. Y. Chan C. K. Y. (2025). From chat to cheat: the disruptive effects of ChatGPT and academic integrity in Hong Kong higher education. SN Comput. Sci. 6:532.

[B28] Hofstede G. (2001). Culture's Consequences: Comparing Values, Behaviors, Institutions and Organizations Across Nations, 2nd Edn. Thousand Oaks, CA: Sage Publications.

[B29] Holmes W. Bialik M. Fadel C. (2019). Artificial Intelligence in Education: Promises and Implications for Teaching and Learning. Boston, MA: Center for Curriculum Redesign.

[B30] Holmes W. Porayska-Pomsta K. Holstein K. Sutherland E. Baker T. Shum S. B. . (2021). Ethics of AI in education: towards a community-wide framework. Int. J. Artificial Intell. Educ. 32, 504–526. doi: 10.1007/s40593-021-00239-1

[B31] Hoodbhoy P. (2020). Pakistan's higher education: the state of the state. Int. Higher Educ. 102, 20–22.

[B32] Hu L. T. Bentler P. M. (1999). Cutoff criteria for fit indexes in covariance structure analysis: conventional criteria versus new alternatives. Struct. Eq. Model. 6, 1–55. doi: 10.1080/10705519909540118

[B33] Hwang G. J. Xie H. Wah B. W. Gašević D. (2020). Vision, challenges, roles and research issues of artificial intelligence in education. Comput. Educ. Artificial Intell. 1:100001. doi: 10.1016/j.caeai.2020.100001

[B34] Khattak Z. I. Khan A. Khan H. Khattak S. S. H. (2012). Quality of higher education in Pakistan. Interdiscipl. J. Contemp. Res. Business 3, 1168–1175.

[B35] Khilji S. E. (2003). To adapt or not to adapt: exploring the role of national culture in HRM—a study of Pakistan. Int. J. Cross Cult. Manage. 3, 109–132. doi: 10.1177/147059580331006

[B36] Klein A. G. Moosbrugger H. (2000). Maximum likelihood estimation of latent interaction effects with the LMS method. Psychometrika 65, 457–474. doi: 10.1007/BF02296338

[B37] Kuh G. D. Kinzie J. Buckley J. A. Bridges B. K. Hayek J. C. (2006). What Matters to Student Success: A Review of the Literature. Washington, DC: National Postsecondary Education Cooperative.

[B38] Kulik J. A. Fletcher J. D. (2016). Effectiveness of intelligent tutoring systems: a meta-analytic review. Rev. Educ. Res. 86, 42–78. doi: 10.3102/0034654315581420

[B39] Lent R. W. Singley D. Sheu H. B. Gainor K. A. Brenner B. R. Treistman D. . (2005). Social cognitive predictors of domain and life satisfaction: exploring the theoretical precursors of subjective well-being. J. Counsel. Psychol. 52, 429–442. doi: 10.1037/0022-0167.52.3.429

[B40] Liu M. Chan C. K. Y. Lo C. K. (2025). Examining the role of generative AI in academic writing assessment: a mixed-methods study in higher education. ESP Rev. English Specific Purposes 7, 146–162.

[B41] Lo N. Chan S. (2025a). Examining the role of generative AI in academic writing assessment: a mixed-methods study in higher education. ESP Rev. 7, 7–45. doi: 10.23191/espkor.2025.7.2.7

[B42] Lo N. Chan S. (2025b). From chat to cheat: the disruptive effects of ChatGPT and academic integrity in Hong Kong Higher Education. SN Comput. Sci. 6:993. doi: 10.1007/s42979-025-04532-x

[B43] Lo N. Chan S. Wong A. (2025). Evaluating teacher, AI, and hybrid feedback in English language learning: impact on student motivation, quality, and performance in Hong Kong. SAGE Open 15:21582440251352907. doi: 10.1177/21582440251352907

[B44] Luckin R. Holmes W. (2016). Intelligence Unleashed: An Argument for AI in Education. London: UCL Knowledge Lab.

[B45] MacKinnon D. P. Lockwood C. M. Hoffman J. M. West S. G. Sheets V. (2002). A comparison of methods to test mediation and other intervening variable effects. Psychol. Methods 7, 83–104. doi: 10.1037/1082-989X.7.1.83 11928892 PMC2819363

[B46] Maxwell S. E. Cole D. A. (2007). Bias in cross-sectional analyses of longitudinal mediation. Psychol. Methods 12, 23–44. doi: 10.1037/1082-989X.12.1.23 17402810

[B47] Maxwell S. E. Cole D. A. Mitchell M. A. (2011). Bias in cross-sectional analyses of longitudinal mediation: partial and complete mediation under an autoregressive model. Multivariate Behav. Res. 46, 816–841. doi: 10.1080/00273171.2011.606716 26736047

[B48] Memon G. R. (2007). Education in Pakistan: the key issues, problems and the key challenges. J. Manage. Social Sci. 3, 47–55.

[B49] Meredith W. (1993). Measurement invariance, factor analysis and factorial invariance. Psychometrika 58, 525–543. doi: 10.1007/BF02294825

[B50] Noel-Levitz R. (2019). Student Satisfaction Inventory. Cedar Rapids, IA: Ruffalo Noel Levitz.

[B51] Nunnally J. C. Bernstein I. (1994). The assessment of reliability. Psychometr. Theor. 3, 248–292.

[B52] Orsini C. Binnie V. I. Jerez O. M. (2016). Motivation as a predictor of dental students' academic achievement: a cross-sectional study. J. Dental Educ. 80, 1337–1344.

[B53] Pike G. R. Smart J. C. Ethington C. A. (2012). The mediating effects of student engagement on the relationships between academic disciplines and learning outcomes: an extension of Holland's theory. Res. Higher Educ. 53, 550–575. doi: 10.1007/s11162-011-9239-y

[B54] Podsakoff P. M. MacKenzie S. B. Lee J. Y. Podsakoff N. P. (2003). Common method biases in behavioral research: a critical review of the literature and recommended remedies. J. Appl. Psychol. 88, 879–903. doi: 10.1037/0021-9010.88.5.879 14516251

[B55] Preacher K. J. Hayes A. F. (2008). Asymptotic and resampling strategies for assessing and comparing indirect effects in multiple mediator models. Behav. Res. Methods 40, 879–891. doi: 10.3758/BRM.40.3.879 18697684

[B56] Qayyum A. Kirkgöz Y. (2021). “Education in emergencies: pandemic pedagogy in Pakistan and Turkey,” in Open(ing) Education: Theory and Practice, eds. D. Conrad and P. Prinsloo (Leiden: Brill), 379–398.

[B57] Rashid T. Asghar H. M. (2016). Technology use, self-directed learning, student engagement and academic performance: examining the interrelations. Comput. Human Behav. 63, 604–612. doi: 10.1016/j.chb.2016.05.084

[B58] Reich J. Ruipérez-Valiente J. A. (2019). The MOOC pivot. Science 363, 130–131. doi: 10.1126/science.aav7958 30630920

[B59] Richardson M. Abraham C. Bond R. (2012). Psychological correlates of university students' academic performance: a systematic review and meta-analysis. Psychol. Bull. 138, 353–387. doi: 10.1037/a0026838 22352812

[B60] Robbins S. B. Lauver K. Le H. Davis D. Langley R. Carlstrom A. (2004). Do psychosocial and study skill factors predict college outcomes? A meta-analysis. Psychol. Bull. 130, 261–288. doi: 10.1037/0033-2909.130.2.261 14979772

[B61] Roll I. Wylie R. (2016). Evolution and revolution in artificial intelligence in education. Int. J. Artificial Intell. Educ. 26, 582–599. doi: 10.1007/s40593-016-0110-3

[B62] Ryan R. M. Deci E. L. (2017). Self-Determination Theory: Basic Psychological Needs in Motivation, Development, and Wellness. New York, NY: The Guilford Press. doi: 10.1521/978.14625/28806

[B63] Ryan R. M. Deci E. L. (2020). Intrinsic and extrinsic motivation from a self-determination theory perspective: definitions, theory, practices, and future directions. Contemp. Educ. Psychol. 61:101860. doi: 10.1016/j.cedpsych.2020.101860

[B64] Saleem S. Mahmood Z. Naz M. (2020). Mental health problems in university students: A prevalence study. FWU J. Soc. Sci. 7, 124–130.

[B65] Schreiner L. A. Nelson D. D. (2013). The contribution of student satisfaction to persistence. J. Coll. Student Retention Res. Theory Prac. 15, 73–111. doi: 10.2190/CS.15.1.f

[B66] Selig J. P. Preacher K. J. (2009). Mediation models for longitudinal data in developmental research. Res. Human Dev. 6, 144–164. doi: 10.1080/15427600902911247

[B67] Selwyn N. (2019). Should Robots Replace Teachers? AI and the Future of Education. Cambridge: Polity Press.

[B68] Sharma H. L. Lingam G. I. Naicker S. (2023). South Asian university students' mental health and wellbeing: an ecological systems theory perspective. Front. Psychol. 14:1002416.

[B69] Sharma K. Papamitsiou Z. Giannakos M. (2021). Building pipelines for educational data using AI and multimodal analytics: a “grey-box” approach. Br. J. Educ. Technol. 52, 1488–1508.

[B70] Sheldon K. M. Krieger L. S. (2007). Understanding the negative effects of legal education on law students: a longitudinal test of self-determination theory. Pers. Social Psychol. Bull. 33, 883–897. doi: 10.1177/0146167207301014 17483395

[B71] Shrout P. E. Bolger N. (2002). Mediation in experimental and nonexperimental studies: new procedures and recommendations. Psychol. Methods 7, 422–445. doi: 10.1037/1082-989X.7.4.422 12530702

[B72] Stupnisky R. H. Renaud R. D. Daniels L. M. Haynes T. L. Perry R. P. (2008). The interrelation of first-year college students' critical thinking disposition perceived academic control, and academic achievement. Res. Higher Educ. 49, 513–530. doi: 10.1007/s11162-008-9093-8

[B73] Syed A. Ali S. S. Khan M. (2018). Frequency of depression, anxiety and stress among the undergraduate physiotherapy students. Pakistan J. Med. Sci. 34, 468–471. doi: 10.12669/pjms.342.12298 29805428 PMC5954399

[B74] Tanveer H. Balz A. Cigdem M. A. Kumar V. (2022). Emerging pedagogies for South Asian education contexts: current practices and future directions. Int. J. Educ. Res. 115:102045. doi: 10.1016/j.ijer.2022.102045

[B75] Tennant R. Hiller L. Fishwick R. Platt S. Joseph S. Weich S. . (2007). The Warwick-Edinburgh mental well-being scale (WEMWBS): development and UK validation. Health Qual. Life Outcomes 5:63. doi: 10.1186/1477-7525-5-63 18042300 PMC2222612

[B76] Thapa P. P. Zayed N. M. Alam M. N. Nitsenko V. S. Rudenko S. Svyrydenko D. (2025). Mediating and moderating role of emotional intelligence between mobile phone use and affective commitment among undergraduate students in academic institutes. Curr. Psychol. 44, 6610–6626. doi: 10.1007/s12144-025-07661-x

[B77] Vallerand R. J. Pelletier L. G. Blais M. R. Briere N. M. Senecal C. Vallieres E. F. (1992). The Academic Motivation Scale: a measure of intrinsic, extrinsic, and amotivation in education. Educ. Psychol. Measure. 52, 1003–1017. doi: 10.1177/0013164492052004025

[B78] Vandenberg R. J. Lance C. E. (2000). A review and synthesis of the measurement invariance literature: suggestions, practices, and recommendations for organizational research. Org. Res. Methods 3, 4–70. doi: 10.1177/109442810031002

[B79] VanLehn K. (2011). The relative effectiveness of human tutoring, intelligent tutoring systems, and other tutoring systems. Educ. Psychol. 46, 197–221. doi: 10.1080/00461520.2011.611369

[B80] Vansteenkiste M. Lens W. Deci E. L. (2006). Intrinsic versus extrinsic goal contents in self-determination theory: another look at the quality of academic motivation. Educ. Psychol. 41, 19–31. doi: 10.1207/s15326985ep4101_4

[B81] Vansteenkiste M. Simons J. Lens W. Sheldon K. M. Deci E. L. (2004). Motivating learning, performance, and persistence: the synergistic effects of intrinsic goal contents and autonomy-supportive contexts. J. Pers. Social Psychol. 87, 246–260. doi: 10.1037/0022-3514.87.2.246 15301630

[B82] Venkatesh V. Davis F. D. (2000). A theoretical extension of the technology acceptance model: four longitudinal field studies. Manage. Sci. 46, 186–204. doi: 10.1287/mnsc.46.2.186.11926

[B83] Walkington C. Sherman M. (2013). Using adaptive learning technologies to personalize instruction: the impact of relevant contexts on performance and learning outcomes. J. Educ. Psychol. 105, 932–945. doi: 10.1037/a0031882

[B84] Wilcox P. Winn S. Fyvie-Gauld M. (2005). “It was nothing to do with the university, it was just the people”: the role of social support in the first-year experience of higher education. Stud. Higher Educ. 30, 707–722. doi: 10.1080/03075070500340036

[B85] Williams L. J. Hartman N. Cavazotte F. (2010). Method variance and marker variables: a review and comprehensive CFA marker technique. Org. Res. Methods 13, 477–514. doi: 10.1177/1094428110366036

[B86] Williamson B. (2021). Making markets through digital platforms: Pearson, edu-business, and the (e)valuation of higher education. Crit. Stud. Educ. 62, 50–66. doi: 10.1080/17508487.2020.1737556

[B87] Zawacki-Richter O. Marín V. I. Bond M. Gouverneur F. (2019). Systematic review of research on artificial intelligence applications in higher education–where are the educators? Int. J. Educ. Technol. Higher Educ. 16:39. doi: 10.1186/s41239-019-0171-0

[B88] Zhai X. Chu X. Chai C. S. Jong M. S. Y. Istenic A. Spector M. . (2021). A review of artificial intelligence (AI) in education from 2010 to 2020. Complexity 2021:8812542. doi: 10.1155/2021/8812542

[B89] Zimet G. D. Dahlem N. W. Zimet S. G. Farley G. K. (1988). The multidimensional scale of perceived social support. J. Pers. Assess. 52, 30–41. doi: 10.1207/s15327752jpa5201_2 2280326

